# Eradication of *Helicobacter pylori* Infection Improves Levodopa Action, Clinical Symptoms and Quality of Life in Patients with Parkinson's Disease

**DOI:** 10.1371/journal.pone.0112330

**Published:** 2014-11-20

**Authors:** Hasriza Hashim, Shahrul Azmin, Hamizah Razlan, Nafisah Wan Yahya, Hui Jan Tan, M. Rizal Abdul Manaf, Norlinah Mohamed Ibrahim

**Affiliations:** 1 Neurology Unit, Department of Medicine, UKM Medical Center, Kuala Lumpur, Malaysia; 2 Department of Community Health, UKM Medical Center, Kuala Lumpur, Malaysia; Glaxo Smith Kline, Denmark

## Abstract

**Background:**

Previous studies have demonstrated a higher prevalence of *Helicobacter pylori (H. pylori)* infection in patients with Parkinson's disease (PD) compared to controls. *H. pylori* infection affects levodopa absorption and its eradication significantly improves clinical response to levodopa. Here, we studied the prevalence of *H. pylori* infection and its eradication effects among our PD patients.

**Methods:**

A prospective study involving idiopathic PD patients on levodopa therapy. ^13^C-urea breath test (UBT) was used to detect *H. pylori*. UBT-positive patients were given standard eradication therapy and followed up at 6 and 12 weeks in an open label single arm design. Repeat UBT was performed at 12 weeks. The UPDRS, PD NMQ, PD NMSS and PDQ-39 were administered at baseline and post-eradication (6 and 12 weeks). Levodopa ‘onset’ time and ON-duration were recorded.

**Results:**

Of 82 patients recruited, 27 (32.9%) had positive UBT. *H. pylori*-positive patients had significantly poorer total UPDRS (p = 0.005) and PDQ39 (p<0.0001) scores compared to *H. pylori*-negative patients. At 12 weeks post-eradication, the mean levodopa onset time shortened by 14 minutes (p = 0.011). The mean ON duration time increased by 56 minutes at week 6 (p = 0.041) and 38 minutes at week 12 (p = 0.035). The total UPDRS scores (p<0.0001), scores for parts II (p = 0.001), III (p<0.0001) and IV (p = 0.009) were significantly better. The total PDQ-39 scores (p = 0.001) and subdomains mobility (p = 0.002), ADL (p = 0.001), emotional well being (p = 0.026) and stigma (p = 0.034) significantly improved. The PD NMSQ did not show significant improvement.

**Conclusions:**

*H. pylori* eradication improved levodopa onset time, ON duration, motor severity and quality of life parameters. Screening and eradication of *H. pylori* is inexpensive and should be recommended in PD patients, particularly those with erratic response to levodopa.

**Trial Registration:**

ClinicalTrials.gov NCT02112812

## Introduction

An association between *H. pylori* and Parkinson's disease (PD) is increasingly recognized. One of the earliest studies conducted in the pre- *H. pylori* era established that duodenal and gastric ulcers were more common in PD patients, and predated the diagnosis of PD by approximately 20 years [1].

The possible role of *H. pylori* in the pathogenesis of PD has also been explored [2], [3], [4], [5]. Immunologically, eradication of *H. pylori* was associated with a reduction in circulating inflammatory markers such as IL-6 and TNF-alpha [6]. This implies that chronic *H. pylori* infection may trigger inflammatory and autoantibody/molecular mimicry mechanisms, which could consequently lead to the destruction of dopaminergic neurons. Interestingly, *H. pylori* has also been shown to have a role in the bio-synthetic route of 1-methyl-4-phenyl-1,2,3,6-tetrahydropyridine (MPTP), which is known to be neurotoxic to dopaminergic neurons [2].

More recent clinical studies confirmed that *H. pylori* infection is indeed more prevalent in PD patients compared to controls [7], [8], [9], [10]. One study showed that the presence of antibodies to *H. pylori* was associated with poorer stride length, which improved following its eradication [3]. As duodenum is the primary site for levodopa absorption, it is postulated that *H. pylori* infection affects levodopa bioavailability by disrupting the duodenal mucosa [11], and producing reactive oxygen species [12], [13], which could inactivate the drug [14].

Despite the established link between *H pylori* infection and PD, detection and eradication of *H. pylori* has not been advocated in the management of PD patients. Guided by our previous findings of a higher prevalence of *H. pylori* infection (48%) among our PD patients compared to controls (21.7%) [10], we conducted this study to determine whether *H. pylori* eradication improved patients symptoms (motor, non-motor and quality of life), and the clinical effectiveness of levodopa.

## Methods

### Study Design

This was a single center prospective study involving consecutive PD patients attending the neurology outpatients tertiary referral clinic in UKM Medical Center, Kuala Lumpur from 1^st^ September 2012 to 28th February 2013. Patients received intervention in a single arm, open-label design. The protocol for this study and CONSORT checklist are available as supporting documents; see Protocol S1 and Checklist S1). The clinical trial registration number is NCT02112812

### Ethics Statement

This study complied with the Declaration of Helsinki, and was conducted in UKM Medical Center, upon receiving approval from the UKM Medical Center Research and Ethics Committee (FF-045-2012). In addition to the information sheet, all patients were verbally explained regarding the study prior to signing the consent form. Written informed consent was obtained from all subjects prior to enrolment, once the investigator made sure that all patients understood the details of the study. Although at the beginning of the study, allowances were made for the next of kin or legal representative to sign the consent form in case patients had mental incapacity, none of the patients enrolled had problems (mentally or physically) which interfered with their ability to give written informed consent.

This study has been retrospectively registered in the ClinicalTrials.gov database. Reason for not registering prior to enrolment of patients was due to the fact that it was not a mandatory requirement to have this study registered, by our approving Research and Ethics Committee board. The authors confirm that all ongoing and related trials for this intervention are registered.

### Participants

Participants were PD patients aged 18 years and above, with Hoehn &Yahr Stages I–IV and on levodopa therapy for at least 1 month. Diagnosis of PD was made by a movement disorder neurologist (NMI). Exclusion criteria were, a diagnosis of secondary parkinsonism or Parkinson's plus syndrome, a history of recent proton pump inhibitors (PPIs) or histamine (H_2_) antagonist use for at least 4 weeks prior to the Urea Breath Test (UBT), a history of recent antibiotics use (less than 6 months), and inability to perform the Urea Breath Test (UBT).

### Clinical measurements

Baseline data such as demography, clinical profile and medication details were recorded into a structured data information sheet via a face-to-face interview. The clinical response to levodopa was recorded by obtaining the levodopa onset time and the ON-duration time in minutes.

***Levodopa onset time*** was defined as the shortest time taken to achieve ON state (improvement in motor or non-motor symptoms) following oral levodopa therapy. ***Levodopa ON-duration time*** was defined as duration the patient remained in the ON state following oral levodopa therapy in minutes. Patients were educated on the above definitions and were asked to record these parameters in a diary at hourly intervals. Patients who did not produce diaries (n = 19) at assessment were asked for an average measure of the above times in the last 72 hours prior to assessment. For those who kept a diary (n = 2), the average time was calculated by the investigator (HH). Motor severity was assessed using UPDRS and Hoehn and Yahr staging, while the patient was ON. Quality of life was measured using the PDQ-39. The non-motor symptoms were screened using the PD NMS Questionnaire (PD NMSQ) and PD NMSS was used to measure the severity of NMS. All assessments were performed by a single examiner (HH) to reduce inter-observer variability.

### Urea Breath Test (UBT)

Diagnosis of *H. pylori* was established with the UBT [15] which used the Non-Dispersive Isotope-Selective InfraRed Spectrometer (NDRIS)[IRIS]. This quantified the ratio of ^13^CO_2_ with our normal breath ^12^CO_2_ in order to measure the current *H. pylori* infection [15].Each patient was given 75 mg of IRIS ^13^C urea mixed in Tang Orange juice. Breath samples were taken at 0 minutes, 10 minutes, 20 minutes and 30 minutes. At each breath, subjects were asked to inhale deeply and hold for 5 seconds and then blow slowly into a bag until it is fully inflated. The breath samples were interpreted using the IRIS Software 2.3 for analysis of Delta (δ) over baseline (DOB) which provided a quantitative indicator of *H. pylori* load, by correlating with the total urease activity in the stomach [16], [17].

DOB value of>4.0 was considered positive, while DOB of <2.5 was considered negative for *H. pylori*. If the value ranged between 2.5 and 4.0, the patient was considered as positive, if they had been on antibiotics, PPI or H_2_ antagonists at four to six weeks prior to testing. Otherwise, the patient was considered as negative.

### Intervention – *Helicobacter pylori* Eradication therapy

Patients with positive UBT were given the recommended [18] eradication therapy in a single arm, open label design protocol by a single investigator (HH). Eradication therapy consisted of oral esomeprazole 40 mg daily, oral clarithromycin 500 mg twice daily and oral amoxicillin 1000 mg twice daily for 1 week. Compliance was ensured with a follow-up telephone call (HH).

Clinical assessments which included disease severity, levodopa onset time, levodopa ON duration time, UPDRS, PDQ 39, PD NMSQ and PD NMS scores were obtained at baseline (Visit 1) and post eradication at 6 weeks(Visit 2) and 12 weeks (Visit 3). All the above measurements were taken during the ON state, following levodopa. A repeat UBT was performed at 3 months following eradication therapy to confirm successful eradication.

## Sample Size and Statistical Analysis

The sample size was calculated based on the following formula:
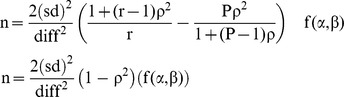



Note: P =  No. of measure before test

r =  No. of measure with test

ρ =  Correlation between pairs of measurement before and after eradication therapy of H. pylori

The calculated number of patients needed in this study was 39 patients. Given the possibility of a 15% drop out rate, a total of 50 patients were recruited. The power of the study was set at 95% with a level of significance of 5%. We screened a total of 120 Parkinson's disease patients using the UBT in order to give us 50 patients with positive UBT.

All data were analyzed using SPSS 20.0 for Windows statistical software package and performed by a statistician. Numerical data were expressed as mean ± standard deviation (SD). Means between *H. pylori* positive and negative were compared using the independent *t-test*. Chi-square test was used to compare categorical variables. Repeated measures ANOVA were used to compare means between three visits. Paired t-tests were used to compare between paired visits; visits (1 & 2), visits (1& 3) and visits (2&3).A p value of <0.05 was taken as being statistically significant.

## Results

**Figure 1:**
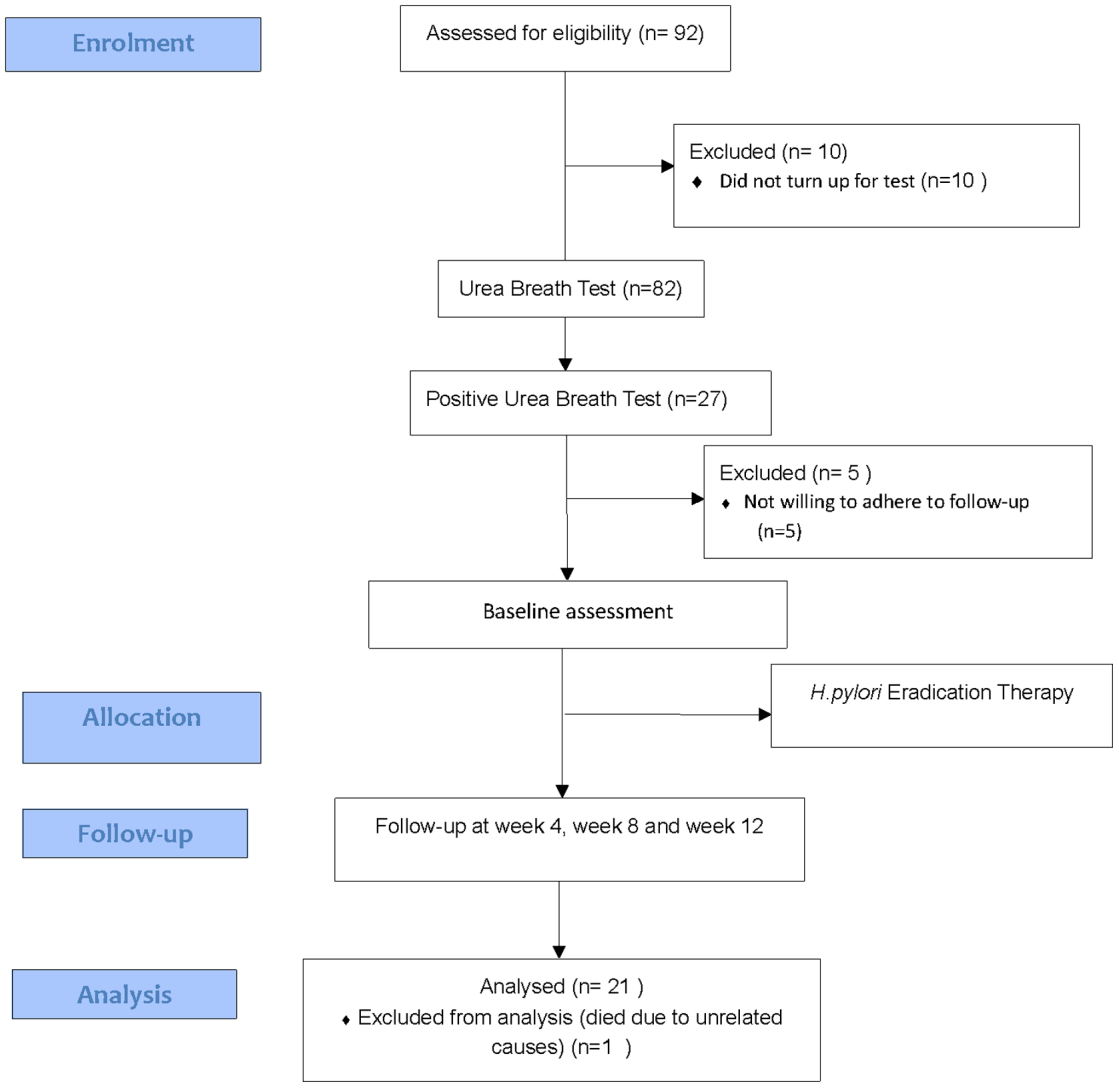
Study Flow Chart.

Ninety-two consecutive PD patients were eligible for the study, but only 82 patients were recruited and underwent the initial UBT. Of these, 27 patients (32.9%) were diagnosed to have *H. pylori* infection. However, 6 UBT-positive patients had to be excluded from the study for various reasons; 5 patients had difficulty coming for follow up visits and 1 patient died from an unrelated event (Figure 1).

Seventy-six patients successfully completed the study; 34 were males (44.7%) and 42 were females (55.3%). The patients comprised of 25 Malay (32.9%), 47 Chinese (61.8%) and 4 Indian (5.3%). The mean age of PD patients in this study was 66.9±8.2 years. The mean duration of PD from diagnosis was 5.16±4.2 years, and mean onset age was 61.7±9.10 years.

### Comparison between *Helicobacter pylori* positive and negative patients

The demographic data and comparative analysis between *H. pylori-* positive and *H. pylori*-negative patients are shown in Table 1. The total UPDRS and subsection 1-4 scores were significantly higher in the *H. pylori*-positive patients compared to the *H. pylori*-negative patients (p = 0.005). Similarly, the PDQ39 scores were higher by 40 points in the *H. pylori*-positive patients compared to the *H. pylori*-negative patients at baseline (p<0.0001) (Table 1).

**Table 1:** Demographic and clinical status of *H. pylori*-positive and *H. pylori*-negative patients.

	*H. pylori* Positive (n = 21)	*H. pylori* Negative (n = 55)	p-value
Age (years)	65.1±9.98	67.5±7.34	0.248θ
Onset Age (years)	59.62±10.61	62.49±8.42	0.221θ
Duration (years)	5 (2.5–8.5)	4 (2–8)	0.427ρ
Male (n,%)	10 (47.6)	24 (43.6)	
Female (n,%)	11 (52.4)	31 (56.4)	
Hoehn &Yahr (n,%)			0.376ρ
Stage 1	0 (0%)	1 (1.8%)	
Stage 2	7 (33.3%)	20 (36.4%)	
Stage 2.5	3 (14.3%)	13 (23.6%)	
Stage 3	7 (33.3%)	13 (23.6%)	
Stage 4	2 (9.5%)	5 (9.1%)	
Stage 5	2 (9.5%)	3 (5.5%)	
Total daily levodopa dose (mg/day)	500 (350–600)	400 (250–600)	0.359 θ
Time to achieve ‘ON’ State (min)	43.1±20.5	40.6±15.9	0.582θ
Duration of the ‘ON’ State (min)	234.3±75.1	287.3±127.8	0.079θ
UPDRS Total Score	85.05±25.48	63.98±29.17	**0.005** θ
PDQ-39 Total Score	80 (58–102)	41 (29–75)	**<0.0001** θ
PDNMS Total Score	55 (40–90.5)	56 (36–84)	0.732θ

θ Independent t-test,

ρ Pearsons Chi-Square.

However, there were no significant differences in the Hoehn & Yahr stages (p = 0.852), total levodopa daily dose (p = 0.605) and total score of PD NMSS questionnaire between the two groups (p = 0.886). There were also no significant differences in the mean age, age of onset, gender and PD duration between the *H. pylori*-positive and *H. pylori-*negative groups.

Based on PD NMSQ, the most common NMS were autonomic dysfunction followed by neuropsychiatric and gastrointestinal symptoms. These NMS were present in more than 50% of our study population. However, there were no differences in the frequencies of any of the NMS or NMS severity between the *H. pylori*-positive and *H. pylori*-negative groups.

### Effects of *H. pylori* eradication on Levodopa action

Based on ANOVA, at 12 weeks following successful *H. pylori* eradication, the levodopa onset time shortened by approximately 14 minutes compared to baseline (43.10±20.52 vs 29.52±8.65; p = 0.011)). Analyses with paired t-tests showed significant improvements between visit 1&3 (43.10±20.52 vs 29.52±8.64; p = 0.014) and visits 2&3 (36.90±14.35 vs 29.52±8.64; p = 0.019), but not between visits 1&2 (p = 0.269) The mean levodopa ON-duration time increased by about 56 minutes at week 6 (visit 2) and 38 minutes at week 12 (visit 3) post eradication but these did not reach statistical significance when analysed using ANOVA (p = 0.06). Further analyses using the paired *t-tests* showed that there were significant improvement in the mean ON duration time when we compared visits 1&2 (234.29±75.07 vs 290.00±142.09; p = 0.041) and visits 1&3 (234.29±75.07 vs 272.86±100.36; p = 0.035, but not between visits 2&3 (p = 0.467) (Table 2).

**Table 2:** Clinical Effects at baseline and following *H. pylori* eradication therapy (n = 21).

	Baseline (1^st^ Visit)	2^nd^ Visit	3^rd^ Visit	p value
Time to achieve ON state (minutes)	43.10±20.52	36.90±14.36	29.52±8.65	0.011
Duration of ON state (minutes)	234.29±75.07	290.0±142.09	272.86±100.36	0.064
PDQ 39 total score	79.52±28.21	65.52±24.66	60.71±25.49	0.001
Mobility	27.38±9.66	24.00±10.96	21.10±10.33	0.002
ADL	15.10±4.93	12.10±5.74	11.05±5.76	0.001
Emotional Well Being	9.67±6.39	7.38±4.69	7.76±5.59	0.026
Stigma	6.14±4.64	4.05±3.11	4.00±3.45	0.034
Social Support	3.29±3.13	2.71±2.72	2.38±2.29	0.187
Cognitive Impairment	7.48±4.11	5.81±3.37	5.57±3.75	0.052
Communication	4.62±3.06	4.43±2.58	3.62±2.20	0.085
Bodily Discomfort	5.86±3.02	5.05±2.50	5.24±2.64	0.202
UPDRS Total Score	85.05±25.48	72.05±23.99	66.71±27.04	<0.0001
Part I	4.90±3.05	4.71±2.26	4.90±2.12	0.763
Part II	21.67±9.46	19.14±8.20	18.43±8.10	0.009
Part III	50.62±13.62	41.43±13.33	37.00±15.56	<0.0001
Part IV	7.86±3.89	6.76±3.90	6.38±4.11	0.005
PD NMSS Total Score	66.14±32.92	65.00±31.69	66.24±29.98	0.454
Domain:				
Cardiovascular including falls	2.29±3.408	2.29±3.58	2.10±3.18	0.715
Sleep/Fatigue	9.24±7.11	9.38±7.42	10.38±7.71	0.336
Mood/Cognition	11.90±8.50	11.95±8.39	12.95±8.04	0.245
Perceptual Problems/Hallucinations	3.29±4.36	2.90±4.00	3.00±3.90	0.275
Attention/Memory	9.05±7.78	8.71±7.61	8.90±7.90	0.552
Gastrointestinal Tract	9.81±5.56	9.71±5.26	8.67± 4.45	0.138
Urinary	8.29±6.66	8.19±6.53	8.10±6.43	0.329
Sexual Function	5.62±3.89	5.62±3.89	5.86±3.94	0.171
Miscellaneous	6.67±3.92	6.24±4.13	6.29±4.19	0.501

All analyses were performed using ANOVA.

### Effects of *H. pylori* eradication on Motor Severity, Non-Motor Symptoms and Severity and Quality of Life

Following eradication, the total UPDRS scores improved significantly by approximately 13 points at week 6 (72.05±0.99) and 20 points (66.71±27.04) by week 12 compared to baseline (85.05±25.48; p<0.0001). Except for Part 1, all components of the UPDRS improved significantly at week 12. The UPDRS-Part II improved by approximately 3 points compared to baseline (21.67±9.46 vs 18.43±8.10; p = 0.009). The UPDRS-Part III scores improved by approximately 13 points at week 12 (50.62±13.62 vs 37.0±15.56; p<0.0001). The UPDRS-Part IV scores improved significantly by 1.5 points (p = 0.005) (Table 2).

Based on the PD NMQ, there were no significant differences in the frequencies of any of the NMS particularly the gastrointestinal symptoms before and after eradication of *H. pylori* infection. However, based on the PD NMS there was a trend for improvement in the gastrointestinal symptom severity post eradication, but not reaching statistical significance.

The total PDQ-39 scores also improved significantly by approximately 19 points following eradication at 12 weeks compared to baseline (79.52±28.21 vs 60.71±25.49; p = 0.001). The domains of PDQ-39 which improved significantly were mobility (27.38±9.70 vs 21.1±10.33; p = 0.002), ADL (15.10±4.93 vs 11.05±5.76; p<0.001), emotional well being (9.67±6.39 vs 7.76±5.59; p = 0.026) and stigma ((6.14±4.64 vs 4.00±3.45; p = 0.034) (Table 2).

### Follow-up UBT

A follow-up UBT was conducted in all the 21 patients at 12 weeks post eradication. All patients were negative for *H. pylori* infection.

## Discussion

We found that as high as 32.9% of our PD patients tested positive for *H. pylori* infection. This is consistent with findings from a previous case-control study conducted in our center involving a different cohort of PD patients, which yielded a prevalence of 48% [10]. On the other hand, studies in patients with other neurological conditions such as epilepsy failed to consistently demonstrate an association with *H. pylori* infection [19], [20], [21]. Despite extensive research in this area, it remains unclear why PD patients have higher propensity to develop this infection.

In addition to the high prevalence, this study showed that *H. pylori* infection affected patients' motor performances adversely, most likely by interfering with levodopa action. We showed that even at baseline, the *H. pylori*-positive patients had much poorer motor scores based on the total UPDRS and subsections I-IV, compared to *H. pylori-negative* patients, despite being matched for duration and stage. From as early as 6 weeks of eradication therapy, there was a significant overall improvement in the motor severity scores by approximately 15%, which was sustained and in fact improved further over the subsequent 6 weeks by 25%. Paralleling the clinical motor improvement, patients also reported significant improvement in their quality of life, for most of the domains tested, particularly ADL which improved by 22%. These quality of life improvements were also observed from as early as 6 weeks. As this study did not have a placebo arm, we were unable to determine to what extent the observed improvement could have been contributed by a placebo effect or the expectation of reward, mediated by striatal dopamine release [22]. Nevertheless, a previous randomized placebo-controlled double-blind study involving a small number of PD patients showed that *H. pylori* eradication improved clinical symptoms and plasma levodopa levels, compared to the placebo group which received only antioxidants. The authors concluded that this improvement was due better levodopa absorption post eradication [23].

In correlating the motor and ADL improvements to levodopa action, we found that eradication of H.pylori significantly improved both the levodopa onset time by approximately 14 minutes at week 12 post eradication, and the duration of ON by approximately 56 minutes at 6 weeks, and 38 minutes at 12 weeks post eradication. Hence, as suggested previously, the clinical improvement following *H. pylori* eradication was most probably due to better levodopa absorption in the gut [8], [9], which translated to an improvement in clinical fluctuations. This notion is supported by a previous study on PD patients which utilized pharmacokinetic sampling in addition to their eradication regime [9]. In that study, eradication of *H. pylori* led to an improvement in intestinal levodopa absorption, measured by the peak plasma levodopa levels, and improvement in motor performance. More recently, a study on the pharmacokinetics (PK) of levodopa in PD patients with motor fluctuations showed that even at baseline, there were differences in the PK parameters of levodopa and 3-OMD depending on the presence of *H. pylori* infection. They showed that the *t_max_* and *C_max_* were lower in *HP* negative patients while the *AUC_o_* was larger in the *HP* positive groups. They concluded that these differences may potentially have important clinical implications to the patient [24]. Their findings may thus help explain the significant differences in the baseline UPDRS and PDQ39 scores between our *H. pylori*- positive and negative patients.

There are many mechanisms by which *H. pylori* infection may affect levodopa absorption. It is suggested that *H. pylori*-related gastritis may interfere with gastric acid secretion [25] by releasing pro inflammatory cytokine interleukin-1β [26], [27], [28]. As levodopa solubility is pH-sensitive, alterations in gastric acidity may affect levodopa absorption. With eradication, there is normalization of gastric acid secretion [29], leading to better levodopa absorption and potentially improved clinical response [26], [29] In support, studies have demonstrated that *H. pylori* eradication augments the plasma levodopa concentration by up to 51% in PD patients [8], [9]. In addition, *H. pylori* may also disrupt gastric myoelectric function [30] leading to problems with gastric motility and alterations in gastric emptying [31], further affecting levodopa absorption adversely. Studies have also suggested that *H. pylori* may in fact utilize levodopa for growth. *H. pylori* was shown to grow faster in levodopa- and noradrenalin-rich culture medium, than in a medium which closely mimicked the normal gastric environment [32]. All these factors may act synergistically to impair intestinal levodopa absorption in vivo. Consequently, these *H. pylori*-positive PD patients are less likely to achieve therapeutic levodopa levels, and are more prone to fluctuations associated with erratic absorption.

While our findings on motor improvement post *H. pylori* eradication are not new, and in fact concur with previous findings [7], [8], [9], [33] it is worth noting that our study was able to correlate motor improvement to significant improvement in quality of life. The total PDQ39 scores improved by 19 points (24%) at the end of the study, suggesting that eradication of *H*.*pylori* led to better quality of life in our patients. Established factors which contribute towards poor quality of life PD include depression [34], disease severity, presence of clinical fluctuations (UPDRS IV) [35], [36] and cognitive impairment [34]. We postulate that the meaningful improvement in the quality of life among our patients was due to better levodopa absorption, as demonstrated by the shortening of the onset time and prolongation of the ON duration time; the net effect being less severe clinical fluctuations.

While the issue of *H. pylori* infection in PD has been studied extensively with positive results post eradication, it appears that the lack of clinical trials involving a large number of patients, somewhat downplays its significance in clinical practice. To date, *H. pylori* testing and eradication has not been advocated for PD patients despite at least three well-designed RCT which has shown positive effects of eradication. We believe the findings of our study complements previously established effects of *H. pylori* eradication in PD patients. We have shown that *H. pylori* eradication not only improved clinical effectiveness of levodopa and motor performance, but also led to robust and meaningful improvement in the quality of life of our patients.

We acknowledge that we are unable to account for placebo effect of treatment, due to our single arm study design. This was mainly due to ethical concerns on the implications of delaying the necessary treatment to our patients who were *H. pylori* positive, if we were to proceed with a randomized controlled trial. Additionally, a blinded assessment would have removed any potential bias in terms of motor scoring. We also acknowledge that sampling of plasma levodopa would have enhanced our findings particularly pertaining to the pharmacokinetics profile of levodopa in relation to the onset time and ON-duration time. Also, since our study was conducted over a short period of time, we were unable to detect any recurrences of infection, as all of our patients remained negative for *H. pylori* at 3 months post eradication. Prospective clinical trials incorporating biomarkers of disease progression and measurable plasma levodopa levels should be conducted in the future to explore the long-term effects of *H. pylori* eradication and the incidence of recurrences.

In conclusion, the findings of this study are of important relevance in the management of patients with PD. We believe that the detection and eradication of *H. pylori* infection should be considered as part of disease management in PD patients, particularly in those experiencing motor fluctuations early in the disease course. It is an inexpensive and practical option for optimizing levodopa therapy in patients experiencing motor fluctuations; thus potentially reducing the need for newer and more costly medications, especially in the less affluent populations.

## Supporting Information

Checklist S1 **CONSORT Checklist.**
(PDF)

Protocol S1 **Trial Protocol.**
(DOC)

## References

[1] Strang RR (1965) The Association of Gastro-Duodenal Ulceration and Parkinson's Disease. The Medical journal of Australia 1: 842–843. 14313339 10.5694/j.1326-5377.1965.tb72277.x

[2] Altschuler E (1996) Gastric Helicobacter pylori infection as a cause of idiopathic parkinson disease and non-arteric anterior optic ischemic neuropathy. Medical Hypotheses 47: 413–414. 8951807 10.1016/s0306-9877(96)90223-6

[3] Bjarnason IT, Charlett A, Dobbs RJ, Dobbs SM, Ibrahim MA, et al (2005) Role of chronic infection and inflammation in the gastrointestinal tract in the etiology and pathogenesis of idiopathic parkinsonism. Part 2: response of facets of clinical idiopathic parkinsonism to Helicobacter pylori eradication. A randomized, double-blind, placebo-controlled efficacy study. Helicobacter 10: 276–287. 16104943 10.1111/j.1523-5378.2005.00330.x

[4] Dobbs RJ, Dobbs SM, Weller C, Bjarnason IT, Oxlade NL, et al (2005) Role of chronic infection and inflammation in the gastrointestinal tract in the etiology and pathogenesis of idiopathic parkinsonism. Part 1: eradication of Helicobacter in the cachexia of idiopathic parkinsonism. Helicobacter 10: 267–275. 16104942 10.1111/j.1523-5378.2005.00331.x

[5] Weller C, Charlett A, Oxlade NL, Dobbs SM, Dobbs RJ, et al (2005) Role of chronic infection and inflammation in the gastrointestinal tract in the etiology and pathogenesis of idiopathic parkinsonism. Part 3: predicted probability and gradients of severity of idiopathic parkinsonism based on H. pylori antibody profile. Helicobacter 10: 288–297. 16104944 10.1111/j.1523-5378.2005.00329.x

[6] Dobbs RJ, Charlett A, Purkiss AG, Dobbs SM, Weller C, et al (1999) Association of circulating TNF-α and IL-6 with ageing and parkinsonism. Acta Neurologica Scandinavica 100: 34–41. 10416510 10.1111/j.1600-0404.1999.tb00721.x

[7] Rees K, Stowe R, Patel S, Ives N, Breen K, et al. (2011) Helicobacter pylori eradication for Parkinson's disease. Cochrane Database Syst Rev 11. 10.1002/14651858.CD008453.pub2 PMC13126632 22071847

[8] Pierantozzi M, Pietroiusti A, Galante A, Sancessario G, Lunardi G, et al (2001) *Helicobacter pylori*-induced reduction of acute levodopa absorption in Parkinson's disease patients. Ann Neurol 50: 686–687. 11706979 10.1002/ana.1267

[9] Pierantozzi M, Pietroiusti A, Brusa L, Galati S, Stefani A, et al (2006) *Helicobacter pylori* eradication and l-dopa absorption in patients with PD and motor fluctuations. Neurology 66: 1824–1829. 16801644 10.1212/01.wnl.0000221672.01272.ba

[10] Nafisah W, Najman A, Hamizah R, Azmin S, Rabani R, et al (2013) high prevalence of Helicobacter pylori infection in Malaysian Parkinson's disease patients. Journal of Parkinsonism and Restless Legs Syndrome 3: 63–67.

[11] Hamlet A, Thoreson AC, Nilsson O, Svennerholm AM, Olbe L, et al (1999) Duodenal *Helicobacter pylori* infection differs in cagA genotype between asymptomatic subjjects and patients with duodenal ulcers. Gastroenterology 116: 259–268. 9922305 10.1016/s0016-5085(99)70121-6

[12] Davies G, Simmonds NJ, Stevens TR, Sheaff MT, Banatvala N, et al (1994) *Helicobacter pylori* stimulates antral mucosa reactive oxygen metabolite production *in vivo* . Gut 35: 179–185. 8307467 10.1136/gut.35.2.179 PMC1374491

[13] Salim AS (1993) The relationship between *Helicobacter pylori* and oxygen derived free radicals in the mechanism of duodenal ulceration. Intern Med 32: 359–364. 8400493 10.2169/internalmedicine.32.359

[14] Kankkunen T, Huupponen I, Lahtinen K, Sundell M, Ekman K, et al (2002) Improved stability and release control of of levodopa and metaraminol using ion-change fibers and transdermal iontophoresis. Eur J Pharm Sci 16: 273–280. 12208457 10.1016/s0928-0987(02)00113-6

[15] Kalach N, Briet F, Raymond J, Benhamou PH, Barbet P, et al (1998) The 13carbon urea breath test for the noninvasive detection of Helicobacter pylori in children: comparison with culture and determination of minimum analysis requirements. J Pediatr Gastroenterol Nutr 26: 291–296. 9523864 10.1097/00005176-199803000-00010

[16] Graham DY, Klein PD (2000) Accurate Diagnosis of Helicobacter pylori: 13C-Urea Breath Test. Gastroenterology Clinics of North America 29: 885–893. 11190073 10.1016/s0889-8553(05)70156-4

[17] Zagari RM, Pozzato P, Martuzzi C, Fuccio L, Martinelli G, et al (2005) 13C-Urea Breath Test to Assess Helicobacter pylori Bacterial Load. Helicobacter 10: 615–619. 16302988 10.1111/j.1523-5378.2005.00358.x

[18] Malfertheiner P, Megraud F, O'Morain CA, Atherton J, Axon ATR, et al (2012) Management of Helicobacter pylori infection—the Maastricht IV/Florence Consensus Report. Gut 61: 646–664. 22491499 10.1136/gutjnl-2012-302084

[19] Asadi-Pooya AA, Dehghani SM, Petramfar P, Emami M, Mahmoodi M (2012) Helicobacter pylori infection in patients with epilepsy. Seizure 21: 21–23. 21903421 10.1016/j.seizure.2011.08.011

[20] Ozturk A, Ozturk CE, Ozdemirli B, Yucel M, Bahçebaşı T (2007) Helicobacter pylori infection in epileptic patients. Seizure 16: 147–152. 17126040 10.1016/j.seizure.2006.10.015

[21] Abdul Razak MR, Tan HJ, Razlan H, Ibrahim NM, Sutan R (2012) Prevalence of Helicobacter pylori infection in epilepsy patients in a teaching hospital in Malaysia. Neurology Asia 17: 3.

[22] Goetz CG, Wuu J, McDermott MP, Adler CH, Fahn S, et al (2008) Placebo response in Parkinson's disease: Comparisons among 11 trials covering medical and surgical interventions. Movement Disorders 23: 690–699. 18228568 10.1002/mds.21894

[23] Pierantozzi M, Pietroiusti A, Brusa L, Galati S, Stefani A, et al (2006) Helicobacter pylori eradication and l-dopa absorption in patients with PD and motor fluctuations. Neurology 66: 1824–1829. 16801644 10.1212/01.wnl.0000221672.01272.ba

[24] Narozanska E, Bialecka M, Adamiak-Giera U, Gawronska-Szklarz B, Soltan W, et al (2014) Pharmacokinetics of Levodopa in Patients With Parkinson Disease and Motor Fluctuations Depending on the Presence of Helicobacter pylori Infection. Clinical Neuropharmacology 37: 96–99 10.1097/WNF.0000000000000037. 24992088 10.1097/WNF.0000000000000037

[25] Feldman M, Cryer B, Lee E (1998) Effects of *Helicobacter pylori* gastritis on gastric secretion in healthy human beings. Am J Physiol 274: G1011–1017. 9696699 10.1152/ajpgi.1998.274.6.G1011

[26] Beales ILP, Calam J (1998) Interleukin 1β and tumour necrosis factor α inhibit acid secretion in cultured rabbit parietalo cells by multiple pathways. Gut 42: 227–234. 9536948 10.1136/gut.42.2.227 PMC1726991

[27] Takashima M, Furuta T, Hanai H, Sugimura H, Kaneko E (2001) Effects of Helicobacter pylori infection on gastric acid secretion and serum gastrin levels in Mongolian gerbils. Gut 48: 765–773. 11358893 10.1136/gut.48.6.765 PMC1728329

[28] El-Omar EM (2001) The importance of interleukin 1β in *Helicobacter pylori* disease. Gut 48: 743–747. 11358884 10.1136/gut.48.6.743 PMC1728311

[29] Annibale B, Di Giulio E, Caruana P, Lahner E, Capurso G, et al (2002) The long-term effects of cure of Helicobacter pylori infection on patients with atrophic body gastritis. Alimentary pharmacology & therapeutics 16: 1723–1731. 12269964 10.1046/j.1365-2036.2002.01336.x

[30] Thor P, Lorens K, Tabor S, Herman R, Konturek J, et al (1996) Dysfunction in gastric myoelectric and motor activity in Helicobacter pylori positive gastritis patients with non-ulcer dyspesia. Journal of physiology and pharmacology: an official journal of the Polish Physiological Society 47: 469. 8877902

[31] Miyaji H, Azuma T, Ito S, Abe Y, Ono H, et al (1999) The effect of Helicobacter pylori eradication therapy on gastric antral myoelectrical activity and gastric emptying in patients with non-ulcer dyspepsia. Alimentary Pharmacology and Therapeutics 13: 1303–1310. 10540044 10.1046/j.1365-2036.1999.00621.x

[32] Doherty NC, Tobias A, Watson S, Atherton JC (2009) The Effect of the Human Gut-Signalling Hormone, Norepinephrine, on the Growth of the Gastric Pathogen Helicobacter pylori. Helicobacter 14: 223–230. 19702852 10.1111/j.1523-5378.2009.00682.x

[33] Lee WY, Yoon WT, Shin HY, Jeon SH, Rhee PL (2008) *Helicobacter pylori* infection and motor fluctuation in patients with Parkinson's disease. Mov Disord 23: 1696–1700. 18649391 10.1002/mds.22190

[34] Schrag A, Jahanshahi M, Quinn N (2000) What contributes to quality of life in patients with Parkinson's disease? Journal of Neurology, Neurosurgery & Psychiatry 69: 308–312. 10.1136/jnnp.69.3.308 PMC1737100 10945804

[35] Chapuis S, Ouchchane L, Metz O, Gerbaud L, Durif F (2005) Impact of the motor complications of Parkinson's disease on the quality of life. Movement Disorders 20: 224–230. 15384126 10.1002/mds.20279

[36] Sławek J, Derejko M, Lass P (2005) Factors affecting the quality of life of patients with idiopathic Parkinson's disease-a cross-sectional study in an outpatient clinic attendees. Parkinsonism & Related Disorders 11: 465–468. 16154794 10.1016/j.parkreldis.2005.04.006

