# Supplementary material for: Eradication of Helicobacter pylori Infection Improves Levodopa Action, Clinical Symptoms and Quality of Life in Patients with Parkinson's Disease
Source: PLoS One. 2014 Nov 20;9(11):e112330. doi: 10.1371/journal.pone.0112330 (PMC4239049; doi:10.1371/journal.pone.0112330)
Supplement: Protocol S1 — Trial Protocol. (DOC) [file pone.0112330.s002.doc]

**____________________________________________________________________________**

**A STUDY ON CLINICAL RESPONSE FOLLOWING ERADICATION THERAPY OF *HELICOBACTER PYLORI* INFECTION IN PARKINSON’S DISEASE**

**CANDIDATE : HASRIZA BINTI HASHIM**

**STUDENT ID : P44489**

**CORE SUPERVISORS:** **PROF DR NORLINAH BT IBRAHIM**

**CONSULTANT NEUROLOGIST
 NEUROLOGY UNIT UKMMC**

**ASSOCIATE PROF DR HAMIZAH BT RAZLAN**

**CONSULTANT GASTROENTEROLOGIST**

**GASTROENTEROLOGY UNIT UKMMC**

**CO SUPERVISORS: ASSOCIATE PROF DR TAN HUI JAN**

**CONSULTANT NEUROLOGIST
 NEUROLOGY UNIT UKMMC**

**DR WAN NUR NAFISAH BT WAN YAHYA**

**CONSULTANT NEUROLOGIST
 NEUROLOGY UNIT UKMMC**

**STATISTICIAN:** **ASSOCIATE PROF NAJIB MAHMOOD RAFEE**

**DEPARTMENT OF COMMUNITY HEALTH UKMMC**

**CHAPTER 1**

**Introduction and Literature Review**

**1.1 Parkinson’s Disease**

Parkinson’s disease (PD) is a chronic progressive neurodegenerative disease caused by the depletion of dopaminergic neurons in the substantia nigra. It is characterised by resting tremor, rigidity, bradykinesia and postural instability. Apart from these motor symptoms, non-motor symptoms (NMS) of PD are becoming increasingly recognised (Table 1.1). The diagnosis of PD is a clinical one based on the UK Parkinson’s Disease Society Brain Bank Clinical Diagnostic Criteria (Appendix I). No diagnostic test exists. However, PD needs to be differentiated from other causes of parkinsonism such as secondary Parkinson or Parkinson Plus (table 1.2).

Epidemiology

Parkinson’s disease is the second most common neurodegenerative disorder after Alzheimer’s disease. The worldwide incidence of PD is 1 in 1000 with a mean age of onset of 55-60 years. The disease is uncommon before the age of 40; and the prevalence increases by about 1% after the age of 60 years^1^ and to 3% in people at the age of 80 and older.

Due to a dearth in studies, the prevalence of PD in Malaysia is largely unknown. However, a study of PD prevalence in Singapore^2^ in 1994 showed the crude prevalence of 0.29% in individuals aged 50 and above. Analysis of prevalence in different age groups showed an increase in prevalence in higher age groups up to 25-fold for 80 and above. Inter-racial prevalence rates analysis in the study also showed no difference among Malays (0.23%), Chinese (0.27%) and Indians (0.27%).

Pathogenesis and Causes

The hallmark of idiopathic PD is degeneration of the dopaminergic neurons pathway at the substantia nigra pars compacta of the midbrain^3^ with widespread presence of cytoplasmic inclusions known as Lewy bodies throughout the central and autonomic nervous system. Lewy bodies are abnormal intracytoplasmic aggregates of α-synuclein present in highly selected neurons. In Braak stages of PD^4^, he observed that deposition of the proteinaceous material started at dorsal motor nucleus and advanced upwards thereon. The process continue to progress and once the motor symptoms developed, patients are already at the later staged of the disease. Braak described 6 stages of PD progression, and the deposition of intracytoplasmic Lewy bodies at substantia nigra occurred at Stage III.

Pesticides and herbicides

Exposure to pesticides or herbicides have long been suggested as a risk factor for PD. 1-methyl-4-phenyl-1,2,3,6-tetrahydropyridine (MPTP), a byproduct of illicit heroin synthesis, is known to induce parkinsonism. Investigators have also made the observation that the herbicide paraquat (N,N′-dimethyl-4,4′-bipyridinium dichloride bore a resemblance to 1-methyl-4–phenylpyridinium (MPP), an active metabolite of MPTP. Rajput *et al^5^* , in a study of PD patients in Canada, found that exposure to pesticides positively correlated with increased incidence of PD-like symptoms. Liou *et al^6^* showed that intracerebral injection of paraquat resulted in loss of dopaminergic neurons and depletion of dopamine in the substantia nigra. A Swedish study found that handling pesticides was associated with increased risk of PD by threefold^7^. Another study in Brazil also showed that chemical exposure increased the risk of PD^8^.

Cigarette smoking, caffeine consumption and alcohol

1. Cigarette smoking and caffeine have been shown to have a protective effect against PD. The largest comprehensive study was conducted in 1992 where a group of 14 436 twins from the National Academy of Sciences-National Research Council World War II Veteran Twins Registry were interviewed. They showed that the risk of PD was inversely associated with number of pack years smoked^9^. Interestingly, cigarette smoking does not show a disease-modifying effect when patients who have already been diagnosed with Parkinson’s started smoking^10^.The negative association between PD and coffee/caffeine intake has been demonstrated consistently. As a part of Honolulu Heart Program^11^, data analysed among 8004 Japanese-American men demonstrated that higher caffeine intake is associated with a significantly lower incidence of PD. Both nicotine and caffeine have been demonstrated to prevent neurostriatal degeneration in mice and primates that have been treated with MPTP/MPP. Caffeine was shown to prevent the development of akinesia when given to these dopamine-depleted mice^12^.There is no significant association between alcohol consumption and PD^13^.

The difference in gender prevalence, where male is more preponderance to develop PD, support the possibility of genetic contributions to IPD. Although the genes responsible for Parkinson’s disease have been found, they are not found in most of PD patients.

Treatment and clinical progression

There is no treatment to reverse the neurological disability in PD. Current treatment are only for symptomatic relief by replenishing dopamine to improve motor function and quality of life. Levodopa is the mainstay of treatment for PD, and it dramatically improves parkinsonian symptoms in the early to moderate stages of the disease. However long term use will cause wearing off symptoms and peak dose dyskinesia^14^. Hence, clinicians choose dopamine agonist such as selegiline or pramipexole, instead to delay the use of levodopa especially in younger patients. Other medications that are used with PD are decarboxylase inhibitor such as carbidopa or benserazide to reduce peripheral degradation of levodopa and monoamine oxygenase inhibitor (MAOI) such as selegiline.

**TABLE 1.1 Non-motor Manifestations of Parkinson’s Disease**

(from Simuni & Sethi, Nonmotor Manifestations of Parkinson’s Disease Ann Neurol 2008;64 (suppl):S65-S80)

Neuropsychiatric symptoms

Depression, apathy, anhedonia, anxiety

Dementia

Impulse control disorders

Hallucinations, delusions (usually medications induced)

Sleep Dysfunction

Disorders of sleep initiation and maintenance

Insomnia, poor sleep efficiency

Primary sleep disorders

Restless legs syndrome,

Periodic legs movement disorder

Sleep apnea (obstructive and central)

Parasomnias

REM sleep disorder

Non-REM sleep-related movement disorders

Vivid dreaming

Excessive Daytime Sleepiness

Autonomic Dysfunction

Bladder dysfunction

Orthostatic hypotension

Hyperhidrosis

Sexual dysfunction

Gastrointestinal Symptoms

Constipation

Hypersalivation

Dysphagia

Sensory symptoms

Pain

Olfactory dysfunction

Visual symptoms (diplopia, vision blurring)

Other symptoms

Fatigue

Weight loss

Weight gain (can be medication induced)

**TABLE 1.2 Parkinson-plus syndrome and Secondary Parkinsonism**

| **Parkinson-plus syndrome** | **Secondary Parkinsonism** |
| --- | --- |
| Multiple-system atrophy  (Olivopontocerebellar atrophy  Shy-Drager syndrome)  Progressive supranuclear palsy  Cortico-basal ganglionic degeneration  Vascular Parkinsonism  Dementia with Lewy Bodies | Wilson’s Disease  Drug induced Parkinsonism   - dopamine antagonist (neuroleptic agents, antiemetics)haloperidol - calcium channel antagonists (flunarizine, cinnarizine) - amiodarone - sodium valproate - lithium   Toxic induced – manganese, carbon monoxide, MPTP  Postencephalitic (eg. Encephalitis lethargica, AIDS encephalitis)  Traumatic  Neoplastic  Genetic (DYT12, Parkin mutation, DYT3, ARJP) |

**1.2 *Helicobacter pylori***

*Helicobacter pylori* is a spiral-shaped, gram-negative bacillus that inhabits the gastric mucosa. It is one of the most common infections found in humans worldwide. It was first discovered by Warren and Marshall in 1982 and was then called *Campylobacter pylori*. By the end of the 1980s there was a recognised association between *H. pylori* and antral gastritis and by early-to-mid 1990s further research showed evidence of a link between chronic *H. pylori* infection and gastric malignancies.

The prevalence of *H. pylori* infection varies worldwide ranging between 20-80%^15^. This variation of prevalence is due to several factors such as age, socioeconomic status and ethnicity. In their review, Pounder and Ng^16^ identified two major patterns of *H. pylori* infection prevalence. The first group showed a high prevalence of *H. pylori* infection before the age of 10 which remained high across all age groups (Appendix II). The second group had a low childhood prevalence which increased with age which occurred in developed countries.

In Malaysia, an overall prevalence of 26-60% was noted. Goh et al^17^ studied the prevalence of *H. pylori* infection in patients undergoing endoscopy in the University Hospital in Kuala Lumpur between 1994-1995. They found that the Indian race had the highest prevalence of *H. pylori* than either Chinese or Malays with seropositivity of 61.8%, 48.5% and 16.4% respectively. A study done by Gurjeet et al^18^ among endoscoped patients in north eastern Peninsular Malaysia in 2003 found a low prevalence of 13.5%. They also found similar differences in racial distribution of prevalence for Malays (6.6%), Chinese (24.1%) and Indians (28.6%). Another study by Uyub^19^ looking at 5,370 healthy blood donors in Northern Peninsula showed a lower prevalence of 14.2%.

Humans are the only known host of *H. pylori*. Its mode of transmission is still not fully understood. However, a hypothesis of transmission via feco-oral contamination has been made based on several studies. *H. pylori* has been cultured from vomitus, diarrhoeal stools, saliva supporting this theory. A study of household members who were exposed to another household member infected with *H. pylori* showed an increase of 4.8 fold risk of infection, further supporting human-to-human transmission via faeco-oral mode. A possibility of contracting via contaminated drinking water is still unproven although the presence of *H. pylori* DNA PCR has been documented in drinking water in Peru^20^ that correlates with a high *H. pylori* infection rate among children from low-income group^21^.

Some identified risk factors for high prevalence in a given subpopulation are overcrowding, type of drinking water, lack of toilet facilities during childhood, lower family income and lower educational level^22^.Cigarette smoking, alcohol and coffee consumption are lifestyle factors that have been studied extensively in relation to *H. pylori* infection. In one cross sectional study, Brenner *et al*^23^ found alcohol consumption to have a protective effect against *H. pylori* infection while coffee drinking has the opposite effect.

*H. pylori* is highly adaptive to survive the acidic gastric environment. It produces a urease enzyme which converts urea into carbon dioxide and ammonia. Their flagelli improves their motility to move deeper across the gastric mucous layer. *H. pylori* have a high affinity for mucosal epithelial cells because of a specific bacterial component found on the surface of the bacteria. Once bound to the gastric mucosa epithelia, they continuously trigger gastric inflammation. Recent studies have suggested that *H. pylori* may invade the cytoplasm of the gastric epithelial cells and are thus able to survive for prolonged periods. This might explain their ability to induce a strong immune response and cause gastric disease. *H. pylori* also induce systemic and mucosal humoral responses. Once contracted, *H. pylori* infection does not resolve spontaneously. Instead, it remains within the stomach wall, continuously stimulating host inflammatory reactions.

*H. pylori* infection has been incriminated as the cause of many conditions such as peptic ulcer disease^24^, atrophic gastritis^25^, gastric MALToma^26, 27^ and gastric adenocarcinoma^28, 29^. There were also several extragastric diseases that were associated with *H.pylori* infection such as hepatocellular carcinoma, cholelithiasis, inflammatory bowel disease, idiopathic thrombocytopeania, chronic urticaria and sideropaenic anemia. Some of these conditions have evidence to show that by eradicating *H. pylori* infection, the associated condition will improve or reduce in risk. For example, in chronic urticaria^30^, eradication of *H. pylori* infection helped to resolve the urticaria. In peptic ulcer, eradication helped reduce the recurrence rate of the ulcer.

Association between *H. pylori* infection and gastric cancer has been well demonstrated. *H. pylori* is already considered as a type I (definite) carcinogen due to the evidence acummulated^31, 32^. The EUROGAST study demonstrated an increased gastric malignancy risk with 100% seroprevalence of *H. pylori* compared with seronegative populations. Seropositivity for *H. pylori* infection was associated with a threefold risk of developing gastric adenocarcinoma^28^.

Diagnosis of *H. pylori*

There are several methods of confirming *H. pylori* infection. Oesophagogastroduodenoscopy (OGDS) in combination with the urease test (e.g. CLO test), is useful to confirm the presence of *H. pylori*. OGDS allows direct visualisation of the gastric mucosa and allows for histopathological examination via biopsy.

A non-invasive method which is useful for noncomplicated cases is the ^13^C-Urea Breath test (UBT), where samples of the patient’s breath are taken at baseline and after drinking the C-13 urea. This method has been validated repetitively^33-36^, and is considered the gold standard. However, it lacks the benefit of viewing of the gastric mucosa and sampling for culture and sensitivity.

Serologic testing to detect *H. pylori* IgG or IgA antibodies is an alternative noninvasive test which boasts a sensitivity of 90-100% but has variable specificity of 76-96%^37, 38^. Other test such as PCR, salivary and urinary assays are available but not widely used due to their lack of practicality and/or poor sensitivity.

Treatment

Treatment eradication for *H. pylori* is highly efficacious^39^. The current recommendation for eradication was formulated at the Maastricht III Consensus Conference in March 2005. Eradication treatment for *H. pylori* consists of a proton pump inhibitor (PPI) oral esomeprazole 40mg twice daily, oral clarithromycin 500mg bd and amoxicillin 1000mg bd (or metronidazole in penicillin allergic patients) for a recommended duration of 7 to 14 days^40^.

**1.3 The association between PD and *H. pylori* infection**

The earliest observation that there is an association between *H. pylori* and PD was in 1966 when Strang^41^ found that gastric and duodenal ulcers were more common in PD patients than their age and gender-matched controls and that these ulcers preceded the diagnosis of PD by up to 20 years. Upon the discovery of *H. pylori* and its association with peptic ulcer disease, numerous studies were conducted to look for a correlation between *H. pylori* and PD.

James Parkinson himself considered that there may be an association between PD and gastrointestinal pathology when he wrote “Although unable to trace the connection by which a disordered state of the stomach and bowels may induce a morbid action in a part of the medulla spinalis, …little hesitation needs be employed before we determine on the probability of such occurrence” (pg 64, The Shaking Palsy)^42^.

A direct causal relationship was hypothesised by Altschuler who proposed that *H. pylori* might play a part in the biosynthetic route for MPTP or MPTP-like substance that have a direct neurotoxic effect on the dopaminergic neurons^43^.

In 1999, Charlett *et al.* observed that cardinal features of PD, namely bradykinesia, rigidity and abnormal posture was significantly more prevalent in siblings of PD patients (p=0.01, p<0.001 and p=0.001, respectively) compared to controls. Positive *H. pylori* serology was found in 70% of PD patients and 63% of their siblings compared to 36-43% of controls. They concluded that familial transmission of *H. pylori* might account for the significantly higher prevalence of parkinsonism in siblings of PD patients.

Chronic inflammation leading to neurotoxicity to dopaminergic neurons

Bjarnason *et al*^44^ found that the presence of antibodies against *H. pylori* correlated with IPD, and eradication showed improvement in the measurement of stride length. There was also a correlation between eradication therapy and reduction in inflammatory markers such as IL-6 and TNF-alpha. They hypothesised that chronic infection leads to underlying chronic inflammatory and autoantibody /molecular mimicry mechanism that leads to destruction of the dopaminergic neurons.

Hirai^45^ studied lipid composition of *H. pylori* and identified three cholesterol glucosidases (CG). Of interest, the similarity of the structure of the CG sterols in *H. pylori* and sterol glucosidase in cycad, which may hypothesized a possibility that *H. pylori* with certain strain that can cause parkinsonism by way of producing similar sterol glucosidase that is neurotoxic^46^. Cycad is a type of seed made into flour that has been known to cause ALS-PD disease in population such as in Guam island^47, 48^ and Kii,Japan^49^. Murine experimental exposure to cycad showed progressive motor and cognitive deterioration. Pathological analysis revealed that they have loss of motor neurones and loss of striatal dopaminergic terminals.

*H. pylori* and levodopa absorption

Levodopa absorption occurs in the duodenum. In advanced PD, gastric motility is impaired and this can delay the transit of levodopa through the pylorus and subsequently, prevent or delay its clinical effect. In the duodenum, any inflammatory pathology can also impair levodopa absorption. *H.pylori* infection affects L-dopa bioavailability via disruption of duodenal mucosa, the site of its absorption and local production of reactive oxygen species, which may inactivate the drug^50^.

Irregularity in levodopa delivery to the brain is common in PD and can lead to erratic response to levodopa and motor fluctuations. This can be attributed to impaired gastrointestinal transit or absorption of the drug.

Pierantozzi *et al.* conducted a study in which PD patients with *H. pylori* infection were randomised into eradication therapy and control groups. Serum levodopa levels were consistently higher in the eradication therapy group compared to controls. The active group was also noted to have prolonged clinical response to levodopa, more “on time” and fewer motor fluctuations^50^

The authors concluded that *H. pylori* infection impairs absorption of levodopa and eradication therapy is a simple and inexpensive means to aid levodopa absorption and improve clinical response.

**CHAPTER 2**

**STUDY JUSTIFICATIONS AND OBJECTIVES**

**Study justifications**

Diagnosing *H.pylori* infection in Parkinson’s patients is important as eradicating *H. pylori* not only reduces the risk of H. *pylori* complications such as duodenal ulcer and gastric carcinoma, but it will also help to improve their motor disability. Previous studies abroad have shown that eradication of *H. pylori* increased levodopa bioavailability as *H. pylori* had been shown to affect its absorption. Conducting this study among Parkinson’s patients in PPUKM will give us data for Malaysian populations.

**Study Objectives**

Primary Objectives:

- To compare the clinical response to L-dopa before and after *H. pylori* eradication therapy in the *H. pylori* infected PD group. (L-dopa ‘onset’ time (minutes) and L-dopa ‘on-time’ duration (minutes) ).

Secondary Objectives:

- To assess clinical motor improvement before and after *H. pylori* eradication therapy in infected PD group, using the UPDRS-III and PDQ39 questionnaires.

**CHAPTER 3**

**STUDY HYPOTHESES**

1. *H. pylori* eradication improves L-dopa ‘onset’ time and prolongs the L-dopa ‘on-time’ duration.
2. PD patients with *H. pylori* infection show clinical improvement in motor disability after eradication therapy of H. *pylori*, assessed using UPDRS-III / PDQ39 questionnaires.

**CHAPTER 4**

**METHODOLOGY**

4.1 Study design and study population.

This is a prospective study which, will be conducted at UKMMC between the months of June 2012 to February 2013. The study population consists of Parkinson’s disease patients on levodopa therapy, who will be consecutively recruited from the neurology outpatient clinic UKMMC. The study will be done after the approval of the *Research Ethics Committee UKMMC*. Patients who fulfill the inclusion and exclusion criteria will be counseled and upon giving informed consent, will be enrolled into the study. Individuals with poor command in English or Bahasa Malaysia will be given explanation with an assistance of an interpreter.

4.2 Patient selection

All known PD patients will be recruited from PPUKM neurology clinic consecutively. They will be assessed for stages and severity of their disease using UPDRS and PDQ39 questionnaires. The following information also will be taken from history: duration of disease, types of treatment and its durations of use, previous use of Proton Pump Inhibitors (PPIs)/H2-antagonist and previous endoscopy, symptoms of gastritis/PUD, other co-morbidities and family histories of Parkinson’s and H pylori infection.

Patients who fulfill the following inclusion criteria will be recruited.

Inclusion Criteria

1. Diagnosis of idiopathic Parkinson’s disease (PD) by a neurologist
2. Age more than 18 years
3. Written informed consent given either by the patient or next of kin
4. Patients on L-DOPA therapy

Exclusion Criteria

1. Patients with a diagnosis of secondary parkinsonism, and Parkinson’s plus syndrome

2. History of recent proton pump inhibitors (PPIs) or Histamine (H_2_) antagonist use for at least 4 weeks prior to the urea breath test

3. History of recent antibiotics use (less than 6 months)

4. Inability to perform urea breath test

4.3 Study Flow Chart

*For subject who is on proton pump inhibitor, if there is no contraindication will be withhold for 4 weeks and given a later date for Urea Breath Test

Sample recruitment

Assessment for eligibility

(Fulfillment of Inclusion criteria)

Informed consent

Patient assessment (L-dopa ‘onset’ time and ‘on-time’ duration, UPDRS-III & PDQ39) and data collection

Positive urea breath test (UBT)

Eradication therapy of H.pylori (oral esomeprazole 40mg od, oral clarithromycin 500mg bd and oral amoxicillin 1000mg bd x 14/7)

Assessment of clinical response to L-Dopa (L-dopa ‘onset’ time and ‘on-time’ duration) & clinical motor improvement using UPDRS-III and PDQ-39 Questionnaire

3/12 later

4.4 Sample size calculation

The sample size iscalculated based on the following formula:

n = 2(sd)^2^ 1 + (r-1)ρ^2^ _ Pρ^2^

f(α,β)

diff^2^ r 1 + (P-1)ρ

= 2 (sd)^2^

1 – ρ^2^

f(α,β)

diff^2^

Note: P = No. of measure before test

r = No. of measure with test

ρ = Correlation between pairs of measurement before and after eradication therapy of *H. pylori*

Ref: Phillips R., Campbell M (1997) Journal Pharmaceutical Statistic 7(2) 215-225

Based on study by Lee WY *et al* in 2008, comparing clinical response to L-dopa before and after *H.pylori* eradication therapy in *H.Pylori* infected PD group, the mean L-dopa ‘onset’ time after *H.Pylori* eradication is 58 minutes (25% improvement from baseline). The calculated number of patients needed in this study is 39 patients. Given the possibility of 10% drop out, a total of 50 patients will be recruited. The power of the study is set at 95% with level of significance of 5%. We will screen a total of 120 patients who were diagnosed Parkinson’s disease using Urea Breath Test (UBT) in order to give us 43 patients with positive UBT. Recruitment of total 50 Parkinson’s patients with H. *pylori* infection positive in this study gives a power of study of 95%.

4.5 Study protocol and study tools

Upon consent, patients will be enquired regarding their background and the data will be recorded (Appendix III). Information regarding sociodemographic characteristics (smoking history, alcohol consumption and coffee consumption), previous antibiotics use, concomitant medical conditions, current medications and any gastrointestinal symptoms will be recorded. Height and weight are measured and recorded into the data collection sheet. The clinical response to L-dopa will be documented (looking at L-dopa ‘onset’ time (minutes) and L-dopa ‘on-time’ duration (minutes)). The severity of their Parkinson’s disease will be assessed using PDQ-39 questionnaires and the Unified Parkinson’s Disease Rating Scale (UPDRS)-III.

PDQ-39 (Appendix IV)

PDQ-39 is the most widely used disease-specific patient completed rating scale in PD. It consists of 39 questions covering eight aspects of quality of life: mobility, activities of daily living (ADL), emotional well being, social stigma, social support, cognition, communications and bodily discomfort. PDQ-39 has been tested for reliability and validity^51^. The maximum total score for PDQ-39 is 156 points signifying a poorer quality of life, with subdivisions for mobility (40), ADL (24), emotional well being (24), social stigma (16), social support (12), cognitive impairment (16), communication (12) and bodily discomfort (12).

The Unified Parkinson’s Disease Rating Scale (UPDRS) (Appendix V)

The UPDRS is a rating scale used to assess the severity of PD. It is useful for clinicians as well as researchers to evaluate the progression of the disease and response to treatment. It is comprised of 4 parts. Part I – Mentation, behaviour and mood, Part II – self evaluation of ADLs, Part III – clinician scored motor evaluation and Part IV – Complications of therapy. The first 3 sections (I to III) are rated with a 5-point scale (0 signifying no disability and 4 signifying maximum disability) while the fourth section is used to evaluate patients with more advanced stage who are on treatment. The maximum score for each section is 16, 52, 108 and 23, respectively, giving a total of 199, being the higher the score the greater the disability from PD.

Urea Breath test protocol (Appendix VI)

Each subject undergoes UBT to diagnose H. pylori infection. UBT is selected as the most appropriate tool for diagnosing *H. pylori* in this study because it is noninvasive, non-radiotoxic and readily available compared to OGDS. Serology testing is cheaper and widely accessible and has been used in many researches locally and internationally. Unfortunately, serology testing has a specificity of 79-90% and sensitivity of 76-84%^38, 52^. Furthermore, patients who are positive for *H. pylori* will remain seropositive for as long as 4 years after its eradication even when UBT has long been negative^53^.

Subjects who are on PPIs or H_2_ blockers will be given a later appointment for UBT after 4 weeks of stopping these medications. Each patient will be given 75 mg of IRIS ^13^C urea mixed in Tang™ Orange juice. Breath sample will be taken at 0 minutes, 10 minutes, 20 minutes and 30 minutes. At each breath, subjects will be asked to inhale deeply and hold for 5 seconds and then blow slowly into a bag until its fully inflated. The breath samples collected will be then analysed using the Non-Dispersive Isotope-Selective InfraRed Spectrometer (NDRIS)[IRIS]. Interpretation of the results are based on the IRIS Software 2.3 for analysis of Delta (δ) over baseline (DOB) (Appendix VII).

Delta over baseline (DOB) APPENDIX VII

Delta (δ) over baseline (DOB) of ^13^C-urea breath test has been shown to correlate with H. pylori severity infection. It correlates significantly with total urease activity in the stomach and hence a quantitative indicator of *H. pylori* load^54, 55^. DOB also has been studied extensively and the value shown to be correlated with bacterial strain virulence^56^, intensity of dyspeptic symptom^57^ and therapy efficacy^58^. There is no standard acceptable cutoff for DOB value to divide severity into stages yet. This is because there are many companies with different ^13^C-UBT protocols with differing ^13^C-urea dosages and timing of breath collections.

The IRIS Infra Red isotope Analyser quantifies the ratio of ^13^CO_2_ with our normal breath ^12^CO_2_ in order to measure the current *H. pylori* current infection. DOB value of more than 4.0 is considered *H. pylori* positive while DOB of less than 2.5 is considered negative *H. pylori*. If all the values fall in between the range 2.5 to 4.0, the patient is considered positive if they have taken antibiotics, PPI or H_2_ antagonist for the last 4 to 6 weeks. Otherwise, patient is considered as negative. The sensitivity and specificity of ^13^C-UBT is 98.3% and 98.6% repectively with a positive predicted value and negative predicted value of ^13^C-UBT is 98.2% and 90% respectively^35^.

Treatment for *H. pylori*

Patients with positive Urea Breath Test (positive for *H. pylori* infection) will be started on eradication therapy of *H. pylori* (oral esomeprazole 40mg twice bd, oral clarithromycin 500mg bd and oral amoxicillin 1000mg bd) for 14 days. This current recommendation was formulated at the Maastricht III Consensus Conference in March 2005 ^40^.

Follow up visit

*H pylori*-infected Parkinsons disease patients will be followed up in the neurology clinic 3 months after eradication therapy of *H pylori*. The clinical response to L-dopa after eradication treatment of *H pylori* will be assessed (looking at L-dopa ‘onset’ time (minutes) and L-dopa ‘on-time’ duration (minutes)). The clinical motor improvement will also be assessed using PDQ-39 questionnaires and the Unified Parkinson’s Disease Rating Scale (UPDRS)-III. Patients will be guided by investigator to answer the all the questionnaires.

4.6 Ethical Consideration

This study will be conducted after the approval of the PPUKM ethics committee. Participants will be explained regarding the procedure and the benefit of the study before they signed the consent form. They are allowed to opt out from the study at any point as they wish.

Appendix I


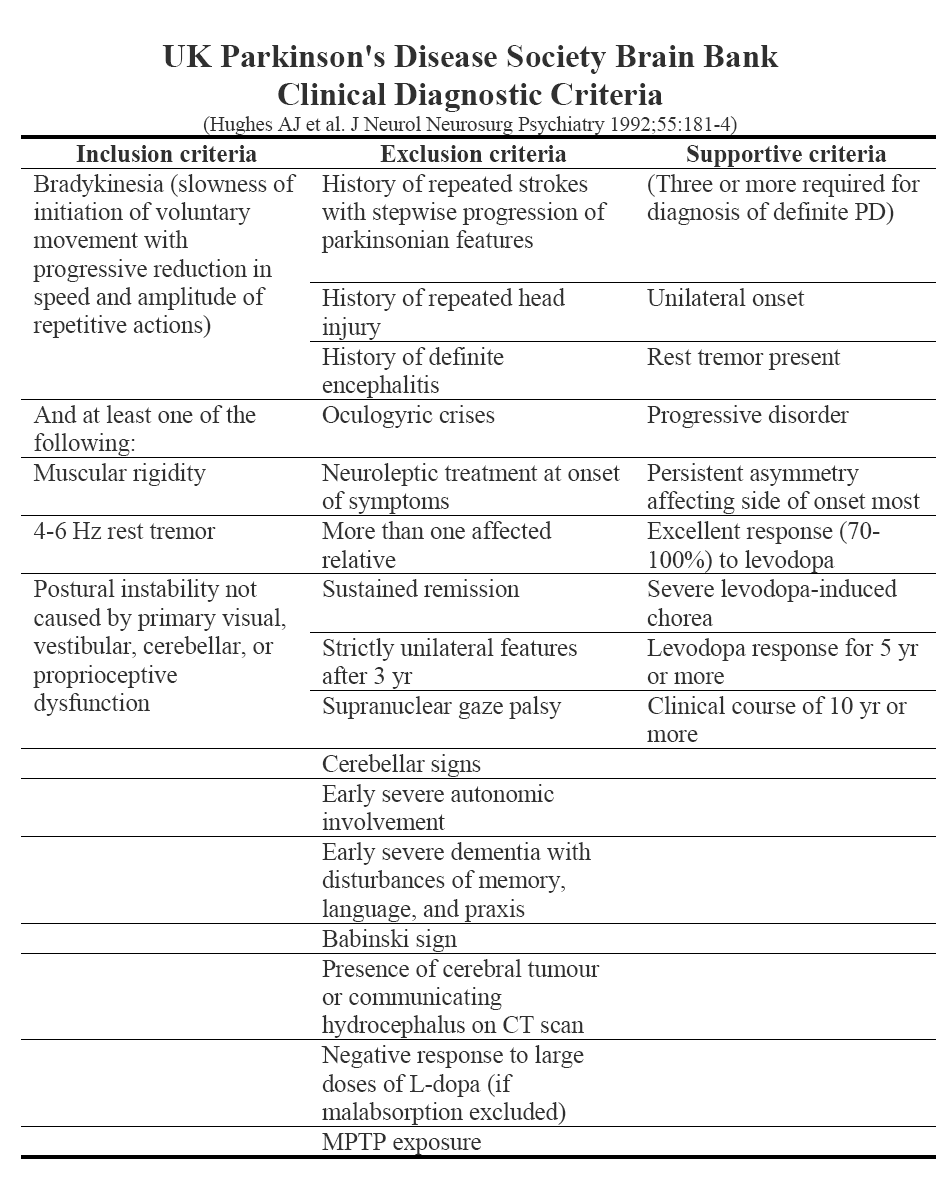


Appendix II

DATA COLLECTION FORM

Subject Initials: Subject #: Date of Visit:

DD MM YR

Paste sticker here Contact Number

A. Checklist:

Parkinson’s Group Control Group

Inclusion Criteria Yes No

1. Diagnosis of idiopathic Parkinson’s disease (PD) by a

neurologist (Hoehn and Yahr Stage I-IV)  

1. Age > 18 and < 90 years old  
2. Written informed consent given either by the patient

or next of kin  

1. Patient on levodopa at least > 1 month  

Exclusion Criteria

1. Patients with a diagnosis of secondary parkinsonism, and

Parkinson’s plus syndrome.  

1. History of recent proton pump inhibitors (PPIs) or histamine (H_2_)

antagonist use for at least 4 weeks prior to the urea breath test  

1. History of recent antibiotics use (less than 6 months)  
2. Inability to perform Urea Breath Test  

Consent  

|  |
| --- |

UBT date

**Urea Breath Test Result positive** 🞏 **negative**🞏

# B. DEMOGRAPHY

**Date of birth Sex**  **Race**

Male Chinese

DD MM YEAR

Female Malay

**Height**: cm Indian

**Weight**: kg Others

_________

**Occupation** :  Professional **Education**  Less than

 Non-professional **level:** primary school

 Unemployed  Secondary School

 Student  University /Colleges

 Master/Postgraduate

and above

**Household Income** < RM 1000

**(per month)** RM 1000-2,500

RM2,500 – 5,000

RM5,000-10,000

>RM10,000

C. PARKINSON’S RISK FACTORS ASSESSMENTS

##

## TOBACCO CONSUMPTION  never

##  occasional

## regular

- ex-smoker

## COFFEE CONSUMPTION  never

##  occasional (<1 cup/day)

 regular (1 cup/day or more)

## ALCOHOL CONSUMPTION  regular drinker__________unit/wk  Non-drinker

## PAST CHEMICAL EXPOSURE  Yes (Details : __________________)

##  No

ANTIBIOTIC USE  Yes (Details : ________________)

- No

FAMILY Hx of:

1. H. Pylori  Yes (Details :_________________)

 No

1. Parkinson’s  Yes (Details _________________)

Disease  No

D. *H. PYLORI* INFECTION ASSESSMENT

## SYMPTOMS: Yes No

## Abdominal pain  

## Heartburn  

## Nausea ± vomiting  

Constipation  

## Melena/Hemetemesis  

## Previous OGDS  

## Previous Diagnosis

## with H pylori   If yes:– Eradication details:

Previous Antibiotic Use   Details :

Drug Allergy   Details :

Concomitant Medical Problems and Medications:

1)______________________________________

2)______________________________________

3)______________________________________

E. Parkinson’s Disease Assessment

Age of Diagnosis of Parkinsons years old

Parkinson’s Medications and dose

1. ____________________________________________________
2. ____________________________________________________
3. ____________________________________________________

Appendix III. Motor Fluctuation Parkinson’s Disease Diary

**EFFECTS OF *HELICOBACTER PYLORI* ERADICATION IN PATIENTS WITH PARKINSON’S DISEASE.**

Name: _________________________________________

Contact No: ____________________________________

Thank you very much for participating in this study.

This is a diary to record your motor status for 72 hours.

The diary should be completed by you. Your caregiver can help in filling up the forms. In case you need any help with your diary completion, please contact Dr. Hasriza Hashim (0173007605)

Please tick only one “status” box for each hourly interval on the page and ensure that no hourly interval is left empty

How to select which box to tick:

1. If your medication was providing benefit with regard to mobility, slowness and stiffness, please tick **“ON”** box for that interval
2. If your medication was providing benefit with regard to mobility, slowness and stiffness, but you were experiencing involuntary twisting, turning movements that *did not* interfere with functioning or *did not* cause meaningful discomfort, please tick **“ON with minor (non-troublesome) dyskinesia”** box for that interval
3. If your medication was providing benefit with regard to mobility, slowness and stiffness, but you were experiencing involuntary twisting, turning movements that *interfered* with functioning or *caused* meaningful discomfort, please tick **“ON with troublesome dyskinesia”** box for that interval
4. If your medication had worn off and was no longer providing benefit with regard to mobility, slowness and stiffness, please tick **“OFF”** box for that interval
5. If you were asleep, please tick **“asleep”** box for that interval
6. In the last column, please tick the appropriate boxes and state the time you took your dose of **levodopa**

Note: Dyskinesias are involuntary twisting and turning movements. These movements are an effect of medication and occur during “ON” period. Tremor is shaking back and forth and is NOT considered dyskinesia. Only YOU (patient) can decide whether the dyskinesia is troublesome or minor.

| **DAY ONE DATE:** | | | | | | |
| --- | --- | --- | --- | --- | --- | --- |
| **Time** | **ON** | **ON with minor dyskinesia** | **ON with troublesome dyskinesia** | **OFF** | **Asleep** | **Levodopa Intake (state exact time)** |
| **0600-0700** |  |  |  |  |  |  |
| **0700-0800** |  |  |  |  |  |  |
| **0800-0900** |  |  |  |  |  |  |
| **0900-1000** |  |  |  |  |  |  |
| **1000-1100** |  |  |  |  |  |  |
| **1100-1200** |  |  |  |  |  |  |
| **1200-1300** |  |  |  |  |  |  |
| **1300-1400** |  |  |  |  |  |  |
| **1400-1500** |  |  |  |  |  |  |
| **1500-1600** |  |  |  |  |  |  |
| **1600-1700** |  |  |  |  |  |  |
| **1700-1800** |  |  |  |  |  |  |
| **1800-1900** |  |  |  |  |  |  |
| **1900-2000** |  |  |  |  |  |  |
| **2000-2100** |  |  |  |  |  |  |
| **2100-2200** |  |  |  |  |  |  |
| **2200-2300** |  |  |  |  |  |  |
| **2300-0000** |  |  |  |  |  |  |
| **0000-0100** |  |  |  |  |  |  |
| **0100-0200** |  |  |  |  |  |  |
| **0200-0300** |  |  |  |  |  |  |
| **0300-0400** |  |  |  |  |  |  |
| **0400-0500** |  |  |  |  |  |  |
| **0500-0600** |  |  |  |  |  |  |

| **DAY TWO DATE:** | | | | | | |
| --- | --- | --- | --- | --- | --- | --- |
| **Time** | **ON** | **ON with minor dyskinesia** | **ON with troublesome dyskinesia** | **OFF** | **Asleep** | **Levodopa Intake (state exact time)** |
| **0600-0700** |  |  |  |  |  |  |
| **0700-0800** |  |  |  |  |  |  |
| **0800-0900** |  |  |  |  |  |  |
| **0900-1000** |  |  |  |  |  |  |
| **1000-1100** |  |  |  |  |  |  |
| **1100-1200** |  |  |  |  |  |  |
| **1200-1300** |  |  |  |  |  |  |
| **1300-1400** |  |  |  |  |  |  |
| **1400-1500** |  |  |  |  |  |  |
| **1500-1600** |  |  |  |  |  |  |
| **1600-1700** |  |  |  |  |  |  |
| **1700-1800** |  |  |  |  |  |  |
| **1800-1900** |  |  |  |  |  |  |
| **1900-2000** |  |  |  |  |  |  |
| **2000-2100** |  |  |  |  |  |  |
| **2100-2200** |  |  |  |  |  |  |
| **2200-2300** |  |  |  |  |  |  |
| **2300-0000** |  |  |  |  |  |  |
| **0000-0100** |  |  |  |  |  |  |
| **0100-0200** |  |  |  |  |  |  |
| **0200-0300** |  |  |  |  |  |  |
| **0300-0400** |  |  |  |  |  |  |
| **0400-0500** |  |  |  |  |  |  |
| **0500-0600** |  |  |  |  |  |  |

| **DAY THREE DATE:** | | | | | | |
| --- | --- | --- | --- | --- | --- | --- |
| **Time** | **ON** | **ON with minor dyskinesia** | **ON with troublesome dyskinesia** | **OFF** | **Asleep** | **Levodopa Intake (state exact time)** |
| **0600-0700** |  |  |  |  |  |  |
| **0700-0800** |  |  |  |  |  |  |
| **0800-0900** |  |  |  |  |  |  |
| **0900-1000** |  |  |  |  |  |  |
| **1000-1100** |  |  |  |  |  |  |
| **1100-1200** |  |  |  |  |  |  |
| **1200-1300** |  |  |  |  |  |  |
| **1300-1400** |  |  |  |  |  |  |
| **1400-1500** |  |  |  |  |  |  |
| **1500-1600** |  |  |  |  |  |  |
| **1600-1700** |  |  |  |  |  |  |
| **1700-1800** |  |  |  |  |  |  |
| **1800-1900** |  |  |  |  |  |  |
| **1900-2000** |  |  |  |  |  |  |
| **2000-2100** |  |  |  |  |  |  |
| **2100-2200** |  |  |  |  |  |  |
| **2200-2300** |  |  |  |  |  |  |
| **2300-0000** |  |  |  |  |  |  |
| **0000-0100** |  |  |  |  |  |  |
| **0100-0200** |  |  |  |  |  |  |
| **0200-0300** |  |  |  |  |  |  |
| **0300-0400** |  |  |  |  |  |  |
| **0400-0500** |  |  |  |  |  |  |
| **0500-0600** |  |  |  |  |  |  |

Appendix III(b).

**KESAN PEMBASMIAN JANGKITAN *HELICOBACTER PYLORI* DI KALANGAN PESAKIT PARKINSON**

Nama: _________________________________________

Nombor telefon: ____________________________________

Terima kasih kerana mangambil bahagian di dalam kajian ini.

Diari ini adalah untuk merekod status pergerakkan anda untuk 72 jam.

Diari ini hendaklah dilengkapi oleh anda. Penjaga anda boleh menolong mengisi ruang yang berkenaan. Sekiranya anda memerlukan bantuan, sila hubungi Dr. Hasriza Hashim (0173007605)

Sila tandakan satu kotak “status” sahaja bagi setiap waktu selang sejam dan pastikan tiada yang dibiarkan kosong.

Bagaimana untuk memilih kotak untuk ditanda:

1. Jika ubat memanfaatkan anda dari segi pergerakkan, perlahan dan kejang, sila tandakan kotak **“ON”** untuk selang waktu itu
2. Jika ubat memanfaatkan anda dari segi pergerakkan, perlahan dan kejang, tetapi anda mengalami pergerakkan memusing, memulas tanpa sengaja yang *tidak* menganggu fungsi atau *tidak* menyebabkan ketidakselesaan yang bermakna, sila tandakan kotak **“ON dengan diskinesia ringan”** untuk selang waktu itu
3. Jika ubat memanfaatkan anda dari segi pergerakkan, perlahan dan kejang, tetapi anda mengalami pergerakkan memusing, memulas tanpa sengaja yang menganggu fungsi atau menyebabkan ketidakselesaan yang bermakna, sila tandakan kotak **“ON dengan diskinesia bermasalah”** untuk selang waktu itu
4. Jika kesan ubat anda telah hilang dan tidak lagi memanfaatkan anda dari segi pergerakkan, perlahan dan kejang, sila tandakan kotak **“OFF”** untuk selang waktu itu.
5. Jika anda tertidur, sila tandakan kotak **“tertidur”** untuk selang waktu itu.
6. Dalam lajur terakhir, sila tandakan kotak yang sesuai dan nyatakan masa pengambilan **levodopa** anda.

NOTA: Diskinesia ialah pergerakkan memusing, memulas tanpa sengaja. Ini adalah kesan ubat dan berlaku semasa keadaan “ON”. Tremor ialah menggeletar dan TIDAK dianggap diskinesia. Hanya ANDA (pesakit) boleh memutuskan sama ada diskinesia tersebut bermasalah atau ringan.

| **HARI PERTAMA TARIKH:** | | | | | | |
| --- | --- | --- | --- | --- | --- | --- |
| **Masa** | **ON** | **ON dengan diskinesia ringan** | **ON dengan diskinesia bermasalah** | **OFF** | **Tertidur** | **Pengambilan Levodopa**  **(Nyatakan masa tepat)** |
| **0600-0700** |  |  |  |  |  |  |
| **0700-0800** |  |  |  |  |  |  |
| **0800-0900** |  |  |  |  |  |  |
| **0900-1000** |  |  |  |  |  |  |
| **1000-1100** |  |  |  |  |  |  |
| **1100-1200** |  |  |  |  |  |  |
| **1200-1300** |  |  |  |  |  |  |
| **1300-1400** |  |  |  |  |  |  |
| **1400-1500** |  |  |  |  |  |  |
| **1500-1600** |  |  |  |  |  |  |
| **1600-1700** |  |  |  |  |  |  |
| **1700-1800** |  |  |  |  |  |  |
| **1800-1900** |  |  |  |  |  |  |
| **1900-2000** |  |  |  |  |  |  |
| **2000-2100** |  |  |  |  |  |  |
| **2100-2200** |  |  |  |  |  |  |
| **2200-2300** |  |  |  |  |  |  |
| **2300-0000** |  |  |  |  |  |  |
| **0000-0100** |  |  |  |  |  |  |
| **0100-0200** |  |  |  |  |  |  |
| **0200-0300** |  |  |  |  |  |  |
| **0300-0400** |  |  |  |  |  |  |
| **0400-0500** |  |  |  |  |  |  |
| **0500-0600** |  |  |  |  |  |  |

| **HARI KEDUA TARIKH:** | | | | | | |
| --- | --- | --- | --- | --- | --- | --- |
| **Masa** | **ON** | **ON dengan diskinesia ringan** | **ON dengan diskinesia bermasalah** | **OFF** | **Tertidur** | **Pengambilan Levodopa**  **(Nyatakan masa tepat)** |
| **0600-0700** |  |  |  |  |  |  |
| **0700-0800** |  |  |  |  |  |  |
| **0800-0900** |  |  |  |  |  |  |
| **0900-1000** |  |  |  |  |  |  |
| **1000-1100** |  |  |  |  |  |  |
| **1100-1200** |  |  |  |  |  |  |
| **1200-1300** |  |  |  |  |  |  |
| **1300-1400** |  |  |  |  |  |  |
| **1400-1500** |  |  |  |  |  |  |
| **1500-1600** |  |  |  |  |  |  |
| **1600-1700** |  |  |  |  |  |  |
| **1700-1800** |  |  |  |  |  |  |
| **1800-1900** |  |  |  |  |  |  |
| **1900-2000** |  |  |  |  |  |  |
| **2000-2100** |  |  |  |  |  |  |
| **2100-2200** |  |  |  |  |  |  |
| **2200-2300** |  |  |  |  |  |  |
| **2300-0000** |  |  |  |  |  |  |
| **0000-0100** |  |  |  |  |  |  |
| **0100-0200** |  |  |  |  |  |  |
| **0200-0300** |  |  |  |  |  |  |
| **0300-0400** |  |  |  |  |  |  |
| **0400-0500** |  |  |  |  |  |  |
| **0500-0600** |  |  |  |  |  |  |

| **HARI KETIGA TARIKH:** | | | | | | |
| --- | --- | --- | --- | --- | --- | --- |
| **Masa** | **ON** | **ON dengan diskinesia ringan** | **ON dengan diskinesia bermasalah** | **OFF** | **Tertidur** | **Pengambilan Levodopa**  **(Nyatakan masa tepat)** |
| **0600-0700** |  |  |  |  |  |  |
| **0700-0800** |  |  |  |  |  |  |
| **0800-0900** |  |  |  |  |  |  |
| **0900-1000** |  |  |  |  |  |  |
| **1000-1100** |  |  |  |  |  |  |
| **1100-1200** |  |  |  |  |  |  |
| **1200-1300** |  |  |  |  |  |  |
| **1300-1400** |  |  |  |  |  |  |
| **1400-1500** |  |  |  |  |  |  |
| **1500-1600** |  |  |  |  |  |  |
| **1600-1700** |  |  |  |  |  |  |
| **1700-1800** |  |  |  |  |  |  |
| **1800-1900** |  |  |  |  |  |  |
| **1900-2000** |  |  |  |  |  |  |
| **2000-2100** |  |  |  |  |  |  |
| **2100-2200** |  |  |  |  |  |  |
| **2200-2300** |  |  |  |  |  |  |
| **2300-0000** |  |  |  |  |  |  |
| **0000-0100** |  |  |  |  |  |  |
| **0100-0200** |  |  |  |  |  |  |
| **0200-0300** |  |  |  |  |  |  |
| **0300-0400** |  |  |  |  |  |  |
| **0400-0500** |  |  |  |  |  |  |
| **0500-0600** |  |  |  |  |  |  |

Appendix III(c).

**KESAN PEMBASMIAN JANGKITAN *HELICOBACTER PYLORI* DI KALANGAN PESAKIT PARKINSON**

姓名: _________________________________________

电话号码: ____________________________________

谢谢您参与这次的研究。

这是一本记录您72小时运动机能的日记。

您需要亲自完成日记。您的护理员可以帮助您填写表格。

请在每半小时的间隔中只在日记页的一个“状态”空格内画勾并且确保完成了所有的一小时间隔。

**如何选择在什么空格里画勾**

1. 如果您的药物治疗对治疗灵动性，迟缓和僵硬有益，请在这半小时间隔的“**起效**”空格中画勾。
2. 如果您的药物治疗对治疗灵动性，迟缓和僵硬有益，但是您在体会不影响身体功能或不会引起重大的不适的偶然的扭曲，旋转运动，请在这半小时间隔的**“伴有轻微的（非棘手运动障碍）”**空格中画勾。
3. 如果您的药物治疗对治疗灵动性，迟缓和僵硬有益, 但是您在体会到影响身体功能或引起重大的不适的偶然的扭曲，旋转运动，请在这半小时间隔的**“伴有运动障碍”**空格中画勾。
4. 如果您的药物治疗效果减弱不再对治疗灵动性，迟缓和僵硬有益, 请在这半小时间隔的**“失效”**空格中画勾。
5. 如果您在熟睡中，请在这半小时间隔的**“熟睡”**空格中画勾。
6. 在最后的竖排中，请在合适的空格勾出您服用levodopa的时间。

注意：运动障碍是偶然的扭曲，旋转运动。这些运动不是药物的效用并且出现在起效时间内。颤动是来回地颤动并且不被认定为运动障碍。

只有您本人（患者）可以决定在任何的半小时时间中出现的运动障碍（偶然的扭曲，旋转运动）是棘手的还是轻微的（不棘手的）。

| **第 一 天 日期 ：** | | | | | | |
| --- | --- | --- | --- | --- | --- | --- |
| **时间** | **起效** | **非棘手**  **运动障碍** | **伴有**  **运动障碍** | **失效** | **熟睡** | **使用 Levodopa**  **写时间** |
| **0600-0700** |  |  |  |  |  |  |
| **0700-0800** |  |  |  |  |  |  |
| **0800-0900** |  |  |  |  |  |  |
| **0900-1000** |  |  |  |  |  |  |
| **1000-1100** |  |  |  |  |  |  |
| **1100-1200** |  |  |  |  |  |  |
| **1200-1300** |  |  |  |  |  |  |
| **1300-1400** |  |  |  |  |  |  |
| **1400-1500** |  |  |  |  |  |  |
| **1500-1600** |  |  |  |  |  |  |
| **1600-1700** |  |  |  |  |  |  |
| **1700-1800** |  |  |  |  |  |  |
| **1800-1900** |  |  |  |  |  |  |
| **1900-2000** |  |  |  |  |  |  |
| **2000-2100** |  |  |  |  |  |  |
| **2100-2200** |  |  |  |  |  |  |
| **2200-2300** |  |  |  |  |  |  |
| **2300-0000** |  |  |  |  |  |  |
| **0000-0100** |  |  |  |  |  |  |
| **0100-0200** |  |  |  |  |  |  |
| **0200-0300** |  |  |  |  |  |  |
| **0300-0400** |  |  |  |  |  |  |
| **0400-0500** |  |  |  |  |  |  |
| **0500-0600** |  |  |  |  |  |  |

| **第 二 天 日期 ：** | | | | | | |
| --- | --- | --- | --- | --- | --- | --- |
| **时间** | **起效** | **非棘手**  **运动障碍** | **伴有**  **运动障碍** | **失效** | **熟睡** | **使用 Levodopa**  **写时间** |
| **0600-0700** |  |  |  |  |  |  |
| **0700-0800** |  |  |  |  |  |  |
| **0800-0900** |  |  |  |  |  |  |
| **0900-1000** |  |  |  |  |  |  |
| **1000-1100** |  |  |  |  |  |  |
| **1100-1200** |  |  |  |  |  |  |
| **1200-1300** |  |  |  |  |  |  |
| **1300-1400** |  |  |  |  |  |  |
| **1400-1500** |  |  |  |  |  |  |
| **1500-1600** |  |  |  |  |  |  |
| **1600-1700** |  |  |  |  |  |  |
| **1700-1800** |  |  |  |  |  |  |
| **1800-1900** |  |  |  |  |  |  |
| **1900-2000** |  |  |  |  |  |  |
| **2000-2100** |  |  |  |  |  |  |
| **2100-2200** |  |  |  |  |  |  |
| **2200-2300** |  |  |  |  |  |  |
| **2300-0000** |  |  |  |  |  |  |
| **0000-0100** |  |  |  |  |  |  |
| **0100-0200** |  |  |  |  |  |  |
| **0200-0300** |  |  |  |  |  |  |
| **0300-0400** |  |  |  |  |  |  |
| **0400-0500** |  |  |  |  |  |  |
| **0500-0600** |  |  |  |  |  |  |

| **第 三 天 日期 ：** | | | | | | |
| --- | --- | --- | --- | --- | --- | --- |
| **时间** | **起效** | **非棘手**  **运动障碍** | **伴有**  **运动障碍** | **失效** | **熟睡** | **使用 Levodopa**  **写时间** |
| **0600-0700** |  |  |  |  |  |  |
| **0700-0800** |  |  |  |  |  |  |
| **0800-0900** |  |  |  |  |  |  |
| **0900-1000** |  |  |  |  |  |  |
| **1000-1100** |  |  |  |  |  |  |
| **1100-1200** |  |  |  |  |  |  |
| **1200-1300** |  |  |  |  |  |  |
| **1300-1400** |  |  |  |  |  |  |
| **1400-1500** |  |  |  |  |  |  |
| **1500-1600** |  |  |  |  |  |  |
| **1600-1700** |  |  |  |  |  |  |
| **1700-1800** |  |  |  |  |  |  |
| **1800-1900** |  |  |  |  |  |  |
| **1900-2000** |  |  |  |  |  |  |
| **2000-2100** |  |  |  |  |  |  |
| **2100-2200** |  |  |  |  |  |  |
| **2200-2300** |  |  |  |  |  |  |
| **2300-0000** |  |  |  |  |  |  |
| **0000-0100** |  |  |  |  |  |  |
| **0100-0200** |  |  |  |  |  |  |
| **0200-0300** |  |  |  |  |  |  |
| **0300-0400** |  |  |  |  |  |  |
| **0400-0500** |  |  |  |  |  |  |
| **0500-0600** |  |  |  |  |  |  |

Appendix IV (a)

**PDQ-39 Q**UESTIONNAIRE

|  | PATIENT ID |  | |  | |  | |  | |
| --- | --- | --- | --- | --- | --- | --- | --- | --- | --- |
|  | DATE OF ASSESSMENT |  |  | |  |  |  | |  |

**EFFECTS OF *HELICOBACTER PYLORI* ERADICATION IN PATIENTS WITH PARKINSON’S DISEASE**

**Please complete the following**

Please tick one box for each question

| No | Due to having Parkinson’s disease, how often during the last month have you…. | Never | Occasionally | Sometimes | Often | Always or cannot do at all |
| --- | --- | --- | --- | --- | --- | --- |
| 1 | Had difficulty doing the leisure activities which you would like to do? |  |  |  |  |  |
| 2 | Had difficulty looking after your home, e.g. DIY, housework, cooking? |  |  |  |  |  |
| 3 | Had difficulty carrying bags of shopping? |  |  |  |  |  |
| 4 | Had problems walking half a mile? |  |  |  |  |  |
| 5 | Had problems walking 100 yards? |  |  |  |  |  |
| 6 | Had problems getting around the house as easily as you would like? |  |  |  |  |  |
| 7 | Had difficulty getting around the public? |  |  |  |  |  |
| 8 | Needed someone else to accompany you when you went out? |  |  |  |  |  |
| 9 | Felt frightened or worried about falling over in public? |  |  |  |  |  |
| 10 | Been confined to the house more than you would like? |  |  |  |  |  |
| 11 | Had difficulty washing yourself? |  |  |  |  |  |
| 12 | Had difficulty dressing yourself? |  |  |  |  |  |
| 13 | Had problems doing up your shoe laces? |  |  |  |  |  |
| 14 | Had problems writing easily? |  |  |  |  |  |
| 15 | Had difficulty cutting up your food? |  |  |  |  |  |
| 16 | Had difficulty holding a drink without spilling it? |  |  |  |  |  |
| 17 | Felt depressed? |  |  |  |  |  |
| 18 | Felt isolated and lonely? |  |  |  |  |  |
| 19 | Felt weepy or tearful? |  |  |  |  |  |
| 20 | Felt angry or bitter? |  |  |  |  |  |
| 21 | Felt anxious? |  |  |  |  |  |
| 22 | Felt worried about your future? |  |  |  |  |  |
| 23 | Felt you had to conceal your Parkinson’s from people? |  |  |  |  |  |
| 24 | Avoided situations which involve eating or drinking in public? |  |  |  |  |  |
| 25 | Felt embarrassed in public due to having Parkinson’s disease? |  |  |  |  |  |
| 26 | Felt worried by other people’s reaction to you? |  |  |  |  |  |
| 27 | Had problems with your close personal relationships? |  |  |  |  |  |
| 28 | Lacked support in the ways you need from your spouse or partner?  *If you do not have a spouse or partner, tick here* 🞏 |  |  |  |  |  |
| 29 | Lacked support in the ways you need from your family or close friends? |  |  |  |  |  |
| 30 | Unexpectedly fallen asleep during the day? |  |  |  |  |  |
| 31 | Had problems with your concentration, e.g. when reading or watching TV? |  |  |  |  |  |
| 32 | Felt your memory was bad? |  |  |  |  |  |
| 33 | Had distressing dreams or hallucinations? |  |  |  |  |  |
| 34 | Had difficulty with your speech? |  |  |  |  |  |
| 35 | Felt unable to communicate with people properly? |  |  |  |  |  |
| 36 | Felt ignored by people? |  |  |  |  |  |
| 37 | Had painful muscle cramps or spasms? |  |  |  |  |  |
| 38 | Had aches and pains in your joints or body? |  |  |  |  |  |
| 39 | Felt unpleasantly hot or cold? |  |  |  |  |  |

Appendix IV (b)

**SOALAN KAJI SELIDIK PDQ-39**

|  | ID PESAKIT |  | |  | |  | |  | |
| --- | --- | --- | --- | --- | --- | --- | --- | --- | --- |
|  | TARIKH PENILAIAN |  |  | |  |  |  | |  |

**KESAN PEMBASMIAN JANGKITAN *HELICOBACTER PYLORI* DI KALANGAN PESAKIT PARKINSON**

**Sila lengkapkan soalan-soalan berikut**

Sila tandakan satu kotak untuk setiap soalan

| No. | Disebabkan oleh penyakit Parkinson, berapa kerap dalam tempoh sebulan yang lepas anda…. | Tidak pernah | Sekali-sekala | Kadang-kadang | Selalu | Sentiasa/ langsung tidak boleh buat |
| --- | --- | --- | --- | --- | --- | --- |
| 1 | Mengalami kesusahan membuat aktiviti lapang yang anda hendak lakukan? |  |  |  |  |  |
| 2 | Mengalami kesusahan mengurus rumah, sebagai contoh kerja rumah, memasak? |  |  |  |  |  |
| 3 | Mengalami kesusahan membawa beg ketika membeli-belah? |  |  |  |  |  |
| 4 | Mengalami masalah berjalan separuh batu? |  |  |  |  |  |
| 5 | Mengalami masalah berjalan 100 ela? |  |  |  |  |  |
| 6 | Mengalami masalah bergerak di sekitar rumah seperti yang anda kehendaki? |  |  |  |  |  |
| 7 | Mengalami masalah bergerak di khalayak ramai? |  |  |  |  |  |
| 8 | Menghendaki seseorang membantu anda keluar dari rumah? |  |  |  |  |  |
| 9 | Merasa takut atau risau akan terjatuh di khalayak ramai? |  |  |  |  |  |
| 10 | Berada di rumah lebih daripada apa yang anda jangkakan atau kehendaki? |  |  |  |  |  |
| 11 | Mengalami masalah membersihkan diri? |  |  |  |  |  |
| 12 | Mengalami masalah memakai baju sendiri? |  |  |  |  |  |
| 13 | Mengalami masalah memakai tali kasut? |  |  |  |  |  |
| 14 | Mengalami masalah menulis dengan senang? |  |  |  |  |  |
| 15 | Mengalami masalah memotong/memakan makanan sendiri? |  |  |  |  |  |
| 16 | Mengalami masalah memegang gelas tanpa tertumpah? |  |  |  |  |  |
| 17 | Merasa murung? |  |  |  |  |  |
| 18 | Merasa terpinggir dan keseorangan? |  |  |  |  |  |
| 19 | Merasa ingin menangis atau menangis? |  |  |  |  |  |
| 20 | Merasa marah atau geram? |  |  |  |  |  |
| 21 | Merasa cemas? |  |  |  |  |  |
| 22 | Merasa takut akan masa hadapan? |  |  |  |  |  |
| 23 | Merasa anda perlu merahsiakan penyakit Parkinson anda daripada diketahui orang? |  |  |  |  |  |
| 24 | Mengelakkan diri daripada suasana yang berkehendakkan makan atau minum di khalayak ramai? |  |  |  |  |  |
| 25 | Merasa malu di khalayak ramai kerana mengidap penyakit Parkinson? |  |  |  |  |  |
| 26 | Merasa bimbang mengenai reaksi orang terhadap anda? |  |  |  |  |  |
| 27 | Mengalami masalah mengenai perhubungan peribadi? |  |  |  |  |  |
| 28 | Kekurangan bantuan di mana anda kehendakinya daripada pasangan?  *Jika anda tidak mempunyai pasangan, sila tanda di sini* 🞏 |  |  |  |  |  |
| 29 | Kekurangan bantuan di mana anda kehendakinya daripada ahli keluarga atau kawan rapat? |  |  |  |  |  |
| 30 | Tidur pada waktu siang tanpa disangka? |  |  |  |  |  |
| 31 | Mengalami masalah memberi tumpuan, sebagai contoh ketika membaca atau menonton TV? |  |  |  |  |  |
| 32 | Merasa ingatan anda teruk? |  |  |  |  |  |
| 33 | Mengalami kesusahan khayalan atau halusinasi? |  |  |  |  |  |
| 34 | Mengalami masalah tentang percakapan? |  |  |  |  |  |
| 35 | Merasa susah untuk berkomunikasi dengan orang lain secara betul? |  |  |  |  |  |
| 36 | Merasa dipinggirkan oleh orang lain? |  |  |  |  |  |
| 37 | Mengalami kesakitan kejang otot atau kaku? |  |  |  |  |  |
| 38 | Mengalami sakit pada sendi atau badan? |  |  |  |  |  |
| 39 | Merasa tidak enak pada ketika panas atau sejuk ? |  |  |  |  |  |

Appendix IV (c)

|  | ID PESAKIT |  | |  | |  | |  | |
| --- | --- | --- | --- | --- | --- | --- | --- | --- | --- |
|  | TARIKH PENILAIAN |  |  | |  |  |  | |  |

**KESAN PEMBASMIAN JANGKITAN *HELICOBACTER PYLORI* DI KALANGAN PESAKIT PARKINSON**

**柏金遜氏症生活質素問卷**

在過去的一個月，柏金遜氏症對你在下列各項的日常生活影有多少：

（每題請記擇最適合的一個答案；完成後請覆查每條問題是否已剔了一個答案）

|  |  | 從來沒有 | 偶然 | 有時候 | 經常 | 所有時候或  完全做不到 |
| --- | --- | --- | --- | --- | --- | --- |
|  |  | １ | ２ | ３ | ４ | ５ |
|  | 做從前喜歡的消遣活動時有困難 |  |  |  |  |  |
|  | 做家居工作（如煮飯，家務）時有困難 |  |  |  |  |  |
|  | 購物後攜帶所購物品時有困難 |  |  |  |  |  |
|  | 步行半哩（大約800米）時有困難 |  |  |  |  |  |
|  | 步行一百碼（大約90米）時有困難 |  |  |  |  |  |
|  | 在家中自由走動時有困難 |  |  |  |  |  |
|  | 在公眾場所內走動時有困難 |  |  |  |  |  |
|  | 外出時需要別人陪伴 |  |  |  |  |  |
|  | 在公眾場所內很或很擔心會跌倒 |  |  |  |  |  |
|  | 留在家中的時間比起自己希望的為長 |  |  |  |  |  |
|  | 替自己沐浴時有困難 |  |  |  |  |  |
|  | 替自己穿衣時有困難 |  |  |  |  |  |
|  | 替自己扣鈕或縛鞋帶時有困難 |  |  |  |  |  |
|  | 要清楚地書寫時有困難 |  |  |  |  |  |
|  | 用刀切食物時有困難 |  |  |  |  |  |
|  | 拿起水杯要保持不倒潟水會有困難 |  |  |  |  |  |
|  | 感到抑鬱 |  |  |  |  |  |
|  | 感到弧單和被隔離 |  |  |  |  |  |
|  | 感覺想哭或流淚 |  |  |  |  |  |
|  | 感到憤怒或苦澀 |  |  |  |  |  |
|  | 感到焦慮 |  |  |  |  |  |
|  | 替自己的將來感到憂慮 |  |  |  |  |  |
|  | 不想讓他人知道你有柏金遜氏症 |  |  |  |  |  |
|  | 盡量避免在公眾場合飲食 |  |  |  |  |  |
|  | 因自己患有柏金遜氏症，在公眾場合會感到尷尬 |  |  |  |  |  |
|  | 為別人對自己患病所作出的反應而感到擔心 |  |  |  |  |  |
|  | 親密的人際關係因患病而出現問題 |  |  |  |  |  |
|  | 缺乏配偶或伴侶所給予的支持  如沒有配偶或伴侶，請在空格內填 √。 |  |  |  |  |  |
|  | 缺乏家庭或摯友所給予的支持 |  |  |  |  |  |
|  | 在日間無故地睡著 |  |  |  |  |  |
|  | 集中精神時有困難（如正有閱讀或觀看電視） |  |  |  |  |  |
|  | 覺得自己記憶力差 |  |  |  |  |  |
|  | 有發惡夢或出現幻覺的情況 |  |  |  |  |  |
|  | 說話時有困難 |  |  |  |  |  |
|  | 覺得自己不能與別人正常地溝通 |  |  |  |  |  |
|  | 覺得被別人忽視 |  |  |  |  |  |
|  | 肌肉有痛性抽筋 |  |  |  |  |  |
|  | 關節或身體部分覺得疼痛 |  |  |  |  |  |
|  | 對外界環境的冷或熱感到很不舒服  （例：進出空氣調節房間） |  |  |  |  |  |

Appendix V(a)

**PARKINSON’S DISEASE NON MOTOR SYMPTOMS QUESTIONNAIRE**

|  | PATIENT ID |  | |  | |  | |  | |
| --- | --- | --- | --- | --- | --- | --- | --- | --- | --- |
|  | DATE OF ASSESSMENT |  |  | |  |  |  | |  |

**EFFECTS OF *HELICOBACTER PYLORI* ERADICATION IN PATIENTS WITH PARKINSON’S DISEASE**

**NON-MOTOR PROBLEMS IN PARKINSON’S DISEASE**

The movement symptoms of Parkinson’s are well known. However, other problems can sometimes occur as part of the condition or its treatment. It is important that the doctor knows about these, particularly if they are troublesome for you.

A range of problems is listed below. Please tick the box ‘Yes’ if you have experienced it **during the past month.** The doctor or nurse may ask you some questions to help decide. If you have **not** experienced the problem in the past month tick the ‘No’ box. You should answer ‘No’ even if you have had the problem in the past but not in the past month.

**Have you experienced any of the following in the last month?**

Yes No

**1.** Dribbling of saliva during the daytime 🞏 🞏

**2.** Loss or change in your ability to taste or smell 🞏 🞏

**3.** Difficulty swallowing food or drink or problems with choking 🞏 🞏

**4.** Vomiting or feelings of sickness (nausea) 🞏 🞏

**5.** Constipation (less than 3 bowel movements a week) or having to strain to pass a stool (faeces) 🞏 🞏

**6.** Bowel (fecal) incontinence 🞏 🞏

**7.** Feeling that your bowel emptying is incomplete after having been to the toilet 🞏 🞏

**8.** A sense of urgency to pass urine makes you rush to the toilet 🞏 🞏

**9.** Getting up regularly at night to pass urine 🞏 🞏

**10.** Unexplained pains (not due to known conditions such as arthritis) 🞏 🞏

**11.** Unexplained change in weight (not due to change in diet) 🞏 🞏

**12.** Problems remembering things that have happened recently or forgetting to do things 🞏 🞏

**13.** Loss of interest in what is happening around you or doing things 🞏 🞏

**14.** Seeing or hearing things that you know or are told are not there 🞏 🞏

**15.** Difficulty concentrating or staying focussed 🞏 🞏

**16.** Feeling sad, ‘low’ or ‘blue’ 🞏 🞏

**17.** Feeling anxious, frightened or panicky 🞏 🞏

**18.** Feeling less interested in sex or more interested in sex 🞏 🞏

**19.** Finding it difficult to have sex when you try 🞏 🞏

**20.** Feeling light headed, dizzy or weak standing from sitting or lying 🞏 🞏

**21.** Falling 🞏 🞏

**22.** Finding it difficult to stay awake during activities such as working, driving or eating 🞏 🞏

**23.** Difficulty getting to sleep at night or staying asleep at night 🞏 🞏

**24.** Intense, vivid dreams or frightening dreams 🞏 🞏

**25.** Talking or moving about in your sleep as if you are ‘acting’ out a dream 🞏 🞏

**26.** Unpleasant sensations in your legs at night or while resting, and a feeling that you need to move 🞏 🞏

**27.** Swelling of your legs 🞏 🞏

**28.** Excessive sweating 🞏 🞏

**29.** Double vision 🞏 🞏

**30.** Believing things are happening to you that other people say are not true 🞏 🞏

All the information you supply through this form will be treated with confidence and will only be used for the purpose or which it has been collected. Information supplied will be used for monitoring purposes. Your personal data will be processed and held in accordance with the Data Protection Act 1998.

**Developed and validated by the International PD Non Motor Group**

Appendix V(b)

**SOALAN GEJALA BUKAN MOTOR PENYAKIT PARKINSON**

|  | ID PESAKIT |  | |  | |  | |  | |
| --- | --- | --- | --- | --- | --- | --- | --- | --- | --- |
|  | TARIKH PENILAIAN |  |  | |  |  |  | |  |

**KESAN PEMBASMIAN JANGKITAN *HELICOBACTER PYLORI* DI KALANGAN PESAKIT PARKINSON**

**MASALAH GEJALA BUKAN MOTOR PENYAKIT PARKINSON**

Gejala motor Parkinson telah diketahui ramai. Akan tetapi, masalah-masalah lain boleh berlaku disebabkan oleh penyakit atau ubat-ubatan. Adalah sesuatu yang mustahak untuk diketahui oleh para doktor, lebih-lebih lagi apabila masalah ini mengganggu hidup anda.

Senarai masalah-masalah di senaraikan seperti di bawah. Sila tanda di kotak “Ya” sekiranya anda mengalaminya **di dalam masa sebulan yang lalu.** Doktor dan jururawat akan bertanyakan soalan untuk membantu anda untuk memilih jawapan. Sekiranya anda **tidak** mengalami masalah di dalam masa sebulan yang lalu, sila tanda ‘Tidak” pada kotak yang disediakan.

Adakah anda pernah mengalami sebarang tanda seperti di bawah ini di dalam sebulan yang lalu ?

YA TIDAK

1. Air liur meleleh di siang hari 🞏 🞏
2. Hilang atau berlaku perubahan terhadap deria rasa atau bau 🞏 🞏
3. Kesukaran menelan makanan atau minuman atau tersedak ketika makan 🞏 🞏
4. Muntah atau mual 🞏 🞏
5. Sembelit (buang air besar kurang dari 3 kali seminggu) atau terpaksa meneran ketika membuang air besar 🞏 🞏
6. Buang air besar tanpa disedari 🞏 🞏
7. Merasa tidak lawas buang air besar selepas ke tandas 🞏 🞏
8. Terasa ingin membuang air kecil menyebabkan anda bergegas ke tandas 🞏 🞏
9. Kerap bangun tidur di waktu malam untuk membuang air kecil 🞏 🞏
10. Sakit yang tidak diketahui puncanya (bukan disebabkan oleh penyakit

seperti sakit sendi tulang) 🞏 🞏

1. Perubahan berat badan (bukan disebabkan oleh perubahan diet) 🞏 🞏
2. Masalah mengingati peristiwa yang baru berlaku atau terlupa untuk membuat sesuatu perkara 🞏 🞏
3. Hilang rasa minat untuk mengetahui apa yang berlaku di sekitar anda atau untuk melakukan sesuatu 🞏 🞏
4. Melihat atau mendengar sesuatu yang anda ketahui atau diberitahu tiada 🞏 🞏
5. Masalah untuk menumpukan perhatian 🞏 🞏
6. Merasa sedih, kecewa atau tertekan 🞏 🞏
7. Merasa bimbang, takut atau panik 🞏 🞏
8. Merasa kurang atau lebih minat terhadap seks 🞏 🞏
9. Mengalami kesukaran untuk melakukan hubungan seks 🞏 🞏
10. Merasa pening, (seperti nak pitam) atau merasa lemah ketika bangun daripada duduk atau baring 🞏 🞏
11. Jatuh 🞏 🞏
12. Senang rasa mengantuk ketika melakukan aktiviti seperti bekerja, memandu, atau makan 🞏 🞏
13. Sukar untuk tidur atau sukar untuk tidur nyenyak di waktu malam 🞏 🞏
14. Mimpi ngeri, menakutkan atau mimpi yang sukar untuk dilupakan 🞏 🞏
15. Mengigau atau meracau semasa tidur 🞏 🞏
16. Rasa tidak selesa pada kaki anda pada waktu malam atau ketika berehat, dan merasa anda perlu bergerak 🞏 🞏
17. Bengkak kaki 🞏 🞏
18. Peluh yang berlebihan 🞏 🞏
19. Satu objek yang kelihatan menjadi dua 🞏 🞏
20. Percaya sesuatu perkara berlaku kepada anda di mana orang lain mengatakan ianya tidak benar 🞏 🞏

Telah disediakan dan disahkan oleh Kumpualan PD Bukan Motor Antarabangsa.

Appendix VI

**EFFECTS OF *HELICOBACTER PYLORI* ERADICATION IN PATIENTS WITH PARKINSON’S DISEASE**


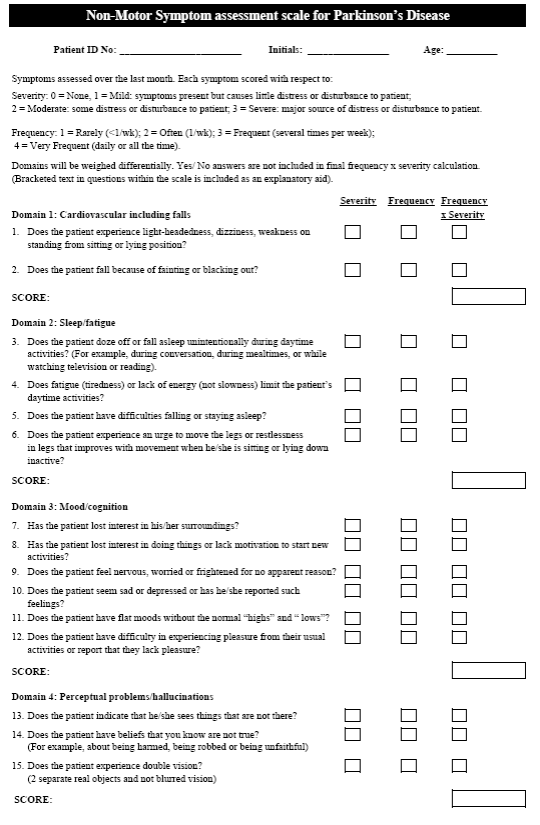


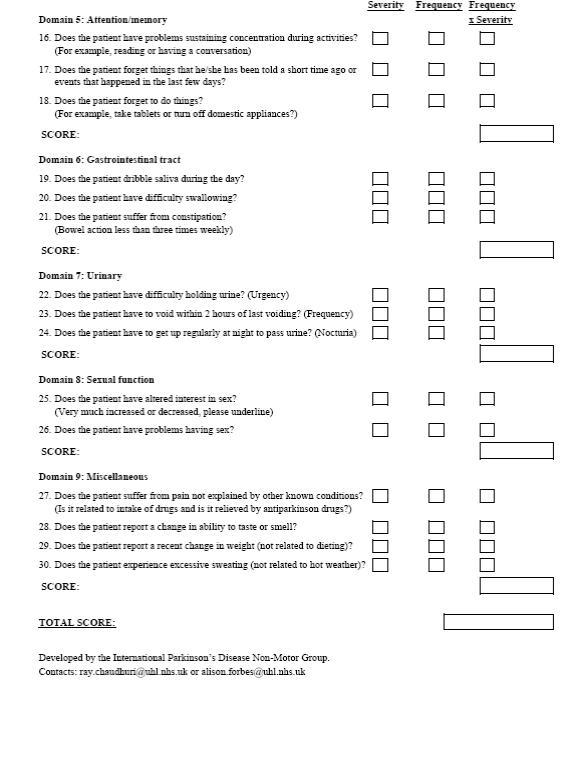


**Appendix VII**

|  | PATIENT ID |  | |  | |  | |  | |
| --- | --- | --- | --- | --- | --- | --- | --- | --- | --- |
|  | DATE OF ASSESSMENT |  |  | |  |  |  | |  |

**EFFECTS OF *HELICOBACTER PYLORI* ERADICATION IN PATIENTS WITH PARKINSON’S DISEASE**

**Unified Parkinson’s Disease Rating Scale**

| PART ONE: MENTATION, BEHAVIOUR AND MOOD | **SCORE** |
| --- | --- |
| ***Intellectual impairment***  0 = None. 1 = Mild. Consistent forgetfulness with partial recollection of events and no other difficulties. 2 = Moderate memory loss, with disorientation and moderate difficulty handling complex problems. Mild but definite impairment of function at home with need of occasional prompting. 3 = Severe memory loss with disorientation for time and often to place. Severe impairment in handling problems. 4 = Severe memory loss with orientation preserved to person only. Unable to make judgments or solve problems. Requires much help with personal care. Cannot be left alone at all. |  |
| *Thought disorder:* (due to Dementia or Drug Intoxication)  0= None. 1= Vivid dreaming. 2="Benign" hallucinations with insight retained. 3=- Occasional to frequent hallucinations or delusions; without insight; could interfere with daily activities. 4= Persistent hallucinations, delusions, or florid psychosis. Not able to care for self. |  |
| *Depression*  0 = Not present. 1= Periods of sadness or guilt greater than normal, never sustained for days or weeks. 2= Sustained depression (1 week or more). 3= Sustained depression with vegetative symptoms (insomnia, anorexia, LOW, loss of interest). 4= Sustained depression with vegetative symptoms and suicidal thoughts or intent. |  |
| *Motivation/Initiative*  0=Normal. 1= Less assertive than usual; more passive. 2= Loss of initiative or disinterest in elective (nonroutine) activities.  3 = Loss of initiative or disinterest in day-to-day (routine) activities.  4= Withdrawn, complete loss of motivation. |  |
| TOTAL SCORE FOR PART ONE |  |
| PART TWO: ACTIVITIES OF DAILY LIVING | **SCORE** |
| *Speech*  0 = Normal. 1= Mildly affected. No difficulty being understood. 2= Moderately affected. Sometimes asked to repeat statements.  3=Severely affected. Frequently asked to repeat statements.  4=Unintelligible most of the time. |  |
| *Salivation*  0= Normal. l= Slight but definite excess of saliva in mouth; may have night time drooling. 2= Moderately excessive saliva; may have minimal drooling. 3= Marked excess of saliva with some drooling. 4= Marked drooling, requires constant tissue or handkerchief. |  |
| *Swallowing*  0=Normal. 1= Rare choking. 2=Occasional choking. 3=Requires soft food. 4= Requires NG tube or gastrotomy feeding. |  |
| *Handwriting*  0=Normal.  1=Slightly slow or small. 2= Moderately slow or small; all words are legible. 3=Severely affected; not all words are legible. 4=The majority of words are not legible. |  |
| *Cutting food and handling utensils*  0=Normal. 1=Somewhat slow and clumsy, but no help needed. 2=Can cut most foods, although clumsy and slow; some help needed.  3=Food must be cut by someone, but can still feed slowly.  4=Needs to be fed. |  |
| *Dressing*  0=Normal. 1=Somewhat slow, but no help needed. 2=Occasional assistance with buttoning, getting arms in sleeves.  3=Considerable help required, but can do some things alone.  4 =Helpless. |  |
| *Hygiene*  0 = Normal. 1 =Somewhat slow, but no help needed. 2=Needs help to shower or bathe; or very slow in hygienic care. *3=* Requires assistance for washing, brushing teeth, combing hair, going to bathroom. 4 =Foley catheter or other mechanical aids. |  |

| *Turning in bed and adjusting bedclothes*  0 =Normal. I =Somewhat slow and clumsy, but no help needed. 2 =Can turn alone or adjust sheets, but with great difficulty. 3 =Can initiate, but not turn or adjust sheets alone. 4 =Helpless. |  | |
| --- | --- | --- |
| *Falling. (unrelated to freezing)*  0 =None. 1 =Rare falling. 2 =Occasionally falls, less than once per day. 3 =Falls an average of once daily. 4 =Falls more than once daily. |  | |
| *Freezing when walking*  0 =None. 1 =Rare freezing when walking; may have start-hesitation. 2 =Occasional freezing when walking. 3= Frequent freezing. Occasionally falls from freezing.  4 =Frequent falls from freezing |  | |
| *Walking*  0 = Normal. I =Mild difficulty. May not swing arms or may tend to drag leg.  2= Moderate difficulty, but requires little or no assistance.  3=Severe disturbance of walking, requiring assistance.  4=Cannot walk at all, even with assistance. |  | |
| *Tremor*  0 =Absent. 1 =Slight and infrequently present. 2 =Moderate; bothersome to patient. 3 =Severe; interferes with many activities. 4 =Marked; interferes with most activities. |  | |
| *Sensory complaints related to parkinsonism*  0= None. I =Occasionally has numbness, tingling, or mild aching. 2 =Frequently has numbness, tingling, or aching; not distressing. 3 =Frequent painful sensations. 4 =Excruciating pain. |  | |
| TOTAL SCORE FOR PART TWO |  | |
| PART THREE: MOTOR EXAMINATION | **SCORE** | |
| Is the patient on medication for treating the symptoms of Parkinson’s Disease?  If yes, how long ago were those medications last taken? _________________________ min  What clinical state is the patient in during this examination? | YES  ON | NO  OFF |
| *Speech*  0= Normal. 1=Slight loss of expression, diction and/ or volume. 2= Monotone, slurred but understandable; moderately impaired.  3= Marked impairment, difficult to understand. 4 =Unintelligible. |  | |
| *Facial expression*  0 =Normal. 1 =Minimal hypomimia, could be normal "Poker Face". 2= Slight but definitely abnormal diminution of facial expression.  3= Moderate hypomimia; lips parted some of the time. 4=Masked or fixed facies with complete loss of facial expression; lips parted 1/4 inch or more. |  | |
| *Tremor at rest*  0 =Absent. 1 =Slight and infrequently present. 2 =Mild in amplitude and persistent. Or moderate in amplitude, but only intermittently present. 3 = Moderate in amplitude and present most of the time. 4= Marked in amplitude and present most of the time. | Face, Lips, Chin | |
|  |  |  |
|  | Rt UE | Lt UE |
|  |  |  |
|  | Rt LE | Lt LE |
|  |  |  |
| *Action or postural tremor of hands*  0 =Absent. *1* =Slight; present with action. 2 =Moderate in amplitude, present with action. 3 =Moderate in amplitude with posture holding as well as action. 4 =Marked in amplitude; interferes with feeding | Rt UE | Lt UE |
|  |  |  |
| *Rigidity: (Judged* on passive movement of major joints with patient relaxed in  sitting position. Cogwheeling to be ignored.)  0 =Absent. 1 =Slight or detectable only when activated by mirror or other movements.  2= Mild to moderate. 3 =Marked, but full range of motion easily achieved. 4 =Severe, range of motion achieved with difficulty.  *Rigidity: (Judged* on passive movement of major joints with patient relaxed in  sitting position. Cogwheeling to be ignored.)  0 =Absent. 1 =Slight or detectable only when activated by mirror or other movements.  2= Mild to moderate. 3 =Marked, but full range of motion easily achieved. 4 =Severe, range of motion achieved with difficulty. | Neck | |
|  |  |  |
|  | Rt UE | Lt UE |
|  |  |  |
|  | Rt LE | Lt LE |
|  |  |  |
| *Finger taps: (Patient* taps thumb with index finger in rapid succession with  widest amplitude possible, each hand separately.)  0= Normal. I =Mild slowing and/or reduction in amplitude. 2= Moderately impaired. Definite and early fatiguing. May have occasional arrests in movement. 3 = Severely impaired. Frequent hesitation in initiating movements or arrests in ongoing movement. 4 = Can barely perform the task. | Rt UE | Lt UE |
|  |  |  |
| *Hand movements: (Patient opens and closes hands in rapid succession with*  *widest amplitude possible, each hand separately.)*  0 =Normal. 1= Mild slowing and/or reduction in amplitude. 2= Moderately impaired. Definite and early fatiguing. May have occasional arrests in movement. 3 =Severely impaired. Frequent hesitation in initiating movements or arrests in ongoing movement. 4 =Can barely perform the task. | Rt UE | Lt UE |
|  |  |  |
| *Rapid alternating movements of hands:* (Pronation-supination movements  of hands, vertically or horizontally, with as large an amplitude as possible,  both hands simultaneously.)  0 =Normal. I =Mild slowing and/or reduction in amplitude. 2= Moderately impaired. Definite and early fatiguing. May have occasional arrests in movement. 3 =Severely impaired. Frequent hesitation in initiating movements or arrests in ongoing movement. 4 =Can barely perform the task | Rt UE | Lt UE |
|  |  |  |
| *26. Leg agility:* (Patient taps heel on ground in rapid succession,  picking up entire leg. Amplitude should be about 3 inches.)  0 = Normal. 1= Mild slowing and/or reduction in amplitude. 2=Moderately impaired. Definite and early fatiguing. May have occasional arrests in movement. 3 =Severely impaired. Frequent hesitation in initiating movements or arrests in ongoing movement. 4 =Can barely perform the task. | Rt LE | Lt LE |
|  |  |  |
| *27. Arising from chair:* (Patient attempts to arise from a straight-back  wood or metal chair with arms folded across chest.)  0 =Normal. I =Slow; or may need more than one attempt. 2 =Pushes self up from arms of seat. 3 =Tends to fall back and may have to try more than one time, but can get up without help. 4 = Unable to arise without help. |  | |

| *Posture*  0 =Normal erect. I =Not quite erect, slightly stooped posture; could be normal for older person. 2=Moderately stooped posture, definitely abnormal; can be slightly leaning to one side. 3 =Severely stooped posture with kyphosis; can be moderately leaning to one side. 4 =Marked flexion with extreme abnormality of posture. |  |
| --- | --- |
| ***29. Gait***  0= Normal. 1= Walks slowly, may shuffle with short steps. but no festination or propulsion. 2= Walks with difficulty, but requires little or no assistance; may have some festination, short steps, or propulsion. 3 = Severe disturbance of gait, requiring assistance. 4=Cannot walk at all, even with assistance. |  |
| *Postural stability:(Response* to sudden posterior displacement produced  by pull on shoulders while patient erect with eyes open and feet slightly apart.  Patient is prepared.)  0=Normal. I= Retropulsion, but recovers unaided. 2=Absence of postural response; would fall if not caught by examiner.  3 =Very unstable, tends to lose balance spontaneously. 4= Unable to stand without assistance. |  |
| *Body bradykinesia and hypokinesia:* (Combining slowness, hesitancy,  decreased armswing, small amplitude, and poverty of movement in general.)  0 = None. 1= Minimal slowness, giving movement a deliberate character; could be normal for some persons. Possibly reduced amplitude. 2= Mild degree of slowness and poverty of movement which is definitely abnormal. Alternatively, some reduced amplitude. 3 = Moderate slowness, poverty or small amplitude of movement. 4 = Marked slowness, poverty or small amplitude of movement. |  |
| TOTAL SCORE FOR PART THREE |  |
| PART FOUR: COMPLICATIONS OF THERAPY |  |
| A. *Dyskinesias*  *Duration: What proportion of the waking day are d^y^skinesias present?*  (Historical information)  0=None 1= 1-25% of day.  2= 26-50% of day.  3= 51-75% of day.  4= 76-100% of day. |  |
| *Disability: How disabling are the dyskinesias? (Historical information;*  *may be modified by office examination.)*  0=Not disabling. 1= Mildly disabling.  2= Moderately disabling.  3=Severely disabling.  4= Completely disabled. |  |
| **34. Painful dyskinesias: How painful are the dyskinesias?** 0= No painful dyskinesias. I =Slight. 2= Moderate. 3=Severe. 4= Marked. |  |
| 35. *Presence of early morning dystonia: (Historical information)* 0= No *1= Yes* |  |
| B. *Clinical Fluctuations*  36. *Are any "off" periods predictable as to timing after a dose of medication?*  *0=No* 1=Yes |  |
| 37. *Are any "off" periods unpredictable as to timing after a dose of medication?*  0=No 1= Yes |  |
| 38. *Do any of the "off periods come on suddenl^y^, e.g., over a few seconds?*  *0=No* 1= Yes |  |
| 39. *What proportion of the waking day is the patient "off" on average?*  0 =None 1= 1-25% of day.  2= 26-50% of day.  3= 51-75% of day.  4= 76-100% of day. |  |
| **C. *Other Complications***  **40. *Does the patient have anorexia, nausea, or vomiting?***  0=No  1 =Yes |  |
| 41. *Does the patient have any sleep disturbances, e.g., insomnia or hyper-somnolence?*  0 = No 1 = Yes |  |
| 42. *Does the patient have symptomatic orthostasis?*  0= No I = Yes |  |
| TOTAL SCORE FOR PART FOUR |  |
| TOTAL UPDRS SCORE |  |

Appendix VIII

**Modified Hoehn and Yahr Staging**

| **Stage** | **Clinical features** |
| --- | --- |
| 0 | No signs of disease |
| 1 | Unilateral disease |
| 1.5 | Unilateral plus axial involvement |
| 2 | Bilateral disease, without impairment of balance |
| 2.5 | Mild bilateral disease, with recovery on pull test |
| 3 | Mild to moderate bilateral disease, some postural instability, physically independent |
| **4** | Severe disability, still able to walk or stand unassisted |
| **5** | Wheelchair bound or bedridden unless aided |

Appendix IX


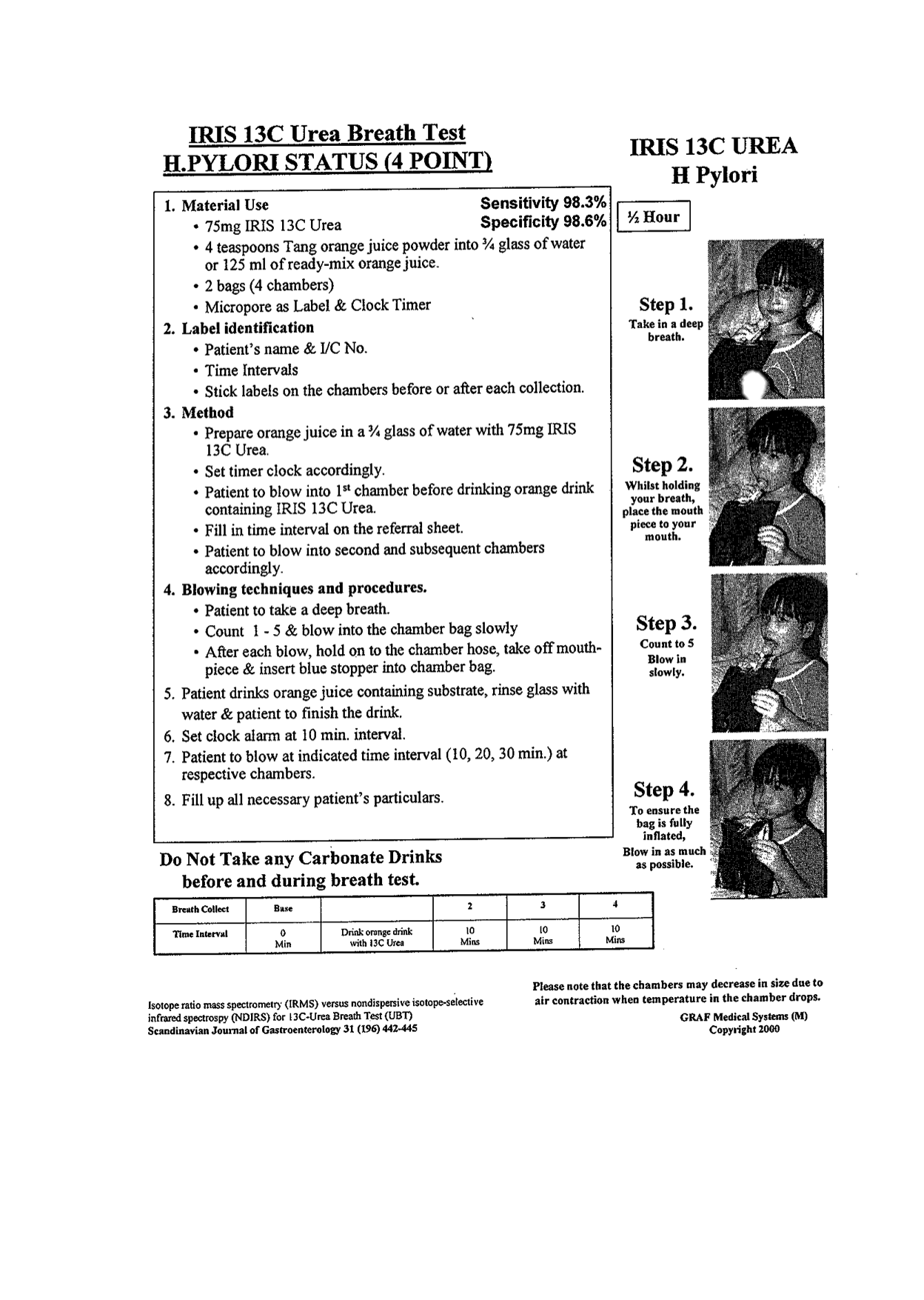


Appendix X

**IRIS analysis of Delta Over Baseline (DOB)**


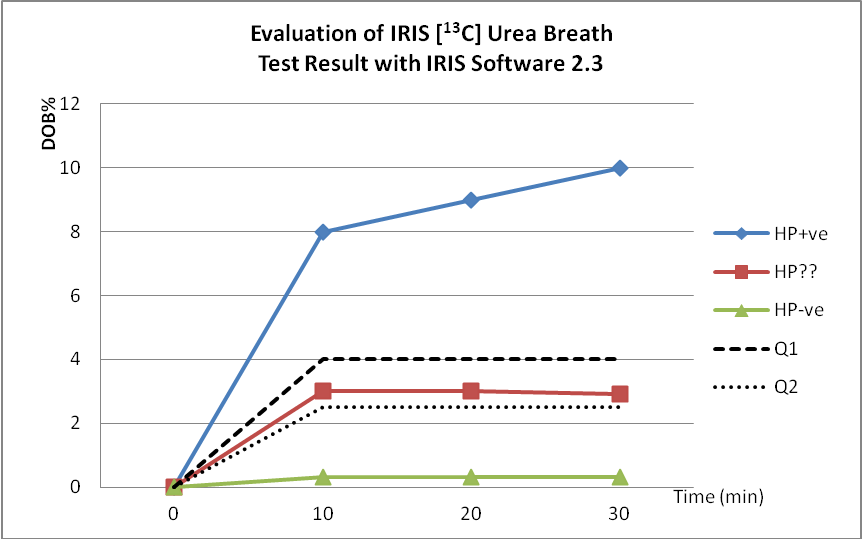


Line above the Q1 is considered HP+ve (DOB >= + 4.0 Delta o/oo)

and line below Q2 indicate patient is HP negative. (DOB <= +2.5 Delta o/oo)

Line between Q1 and Q2 (DOB Range 2.5 – 4.0 Delta o/oo) would indicate H pylori Positive if the patient has been administered with antibiotics, PPP or H2 Blockers in the last 4-6 weeks. Otherwise the patient is *H. pylori* negative.

IRIS Infra Red Isotope Analysis

IRIS Infra Red Isotope Analyser quantifies the ratio of CO_2_ with our normal breath CO_2_ in order to measure the *H. pylori* current infection

Appendix XI(a)

**PATIENT INFORMATION SHEET**

**EFFECTS OF *HELICOBACTER PYLORI* ERADICATION IN PATIENTS WITH PARKINSON’S DISEASE**

(Investigators: Dr. Hasriza Hashim, Prof. Datin Dr. Norlinah Mohamed Ibrahim, Prof. Madya Dr. Hamizah Razlan, Dr Wan Nafisah Wan Yahya, Prof Dr. Tan Hui Jan).

You are invited to take part in this study because you have a Parkinson’s disease (PD)

***Introduction:*** PD is a condition characterised by movement problems such as slowness, tremor and rigidity, as well as non-movement symptoms. Many patients experience abdominal symptoms such as nausea, bloatedness, abdominal discomfort, constipation and weight loss. A few studies suggested that *Helicobacter pylori (H. Pylori)* infection of the stomach occurs frequently in PD.

***Why are we doing this study?*** We would like to determine the clinical response to levodopa therapy and concomitant clinical benefits including both motor and non-motor disability following eradication of *H. Pylori* infection. Better understanding of this issue will help to improve the care of patients with PD.

***What procedures are involved?*** If you agree to participate in this study, you will need to sign an informed consent form. All participants will be asked questions relating to their health and PD-related problems. Trained clinicians will assess the severity of your PD by clinical examination and by using 4 sets of questionnaires (UPDRS, PDQ 39, PD NMSQ, PD NMSS). After an overnight fast, participants will undergo a urea breath tests (UBT) in the same morning to detect *H. Pylori* infection. Patients who are diagnosed to have *H. Pylori* infection will be given antibiotics to eradicate *H.* Pylori and followed up in the next 2 visits (at 6 weeks and 12 weeks post eradication therapy) and assessed clinically.

***What are the risks and safety of the procedures?*** The breath tests already performed on a routine clinical basis, is simple and safe procedure.

***What are the benefits?*** Participants found to be positive for *H. Pylori* infection will be offered appropriate antibiotic treatment for the infection.

***Confidentiality.*** Your identity and examination results will be strictly confidential.

***You are free to withdraw*.** Your participation in this study is strictly voluntary and you can withdraw from the study at any time, without giving any reason, by speaking with your study doctor. Should you decide not to take part in this study, your treatment will carry on as usual.

***Contact persons.*** Please feel free to contact the study doctor if you have any further queries:

Dr Hasriza Hashim, Klinik Perubatan 2, Universiti Kebangsaan Malaysia Medical Centre (UKMMC), Tel No: 03-91455555 ext 7300

Appendix XI(b)

**LAMPIRAN MAKLUMAT PESAKIT**

KAJIAN BERKAITAN KESAN PEMBASMIAN JANGKITAN *HELICOBACTER PYLORI* DI KALANGAN PESAKIT PARKINSON.

(Penyiasat: Dr. Hasriza Hashim, Prof Datin Dr. Norlinah Mohamed Ibrahim, Prof. Madya Dr. Hamizah Razlan, Dr Wan Nafisah Wan Yahya, Prof Dr. Tan Hui Jan.)

Anda dijemput untuk menyertai kajian ini, sama ada sebab anda mempunyai penyakit Parkinson.

***Pengenalan:*** Penyakit Parkinson (PD) merupakan satu penyakit yang berunsurkan masalah pergerakkan seperti kelembapan pergerakkan, kegeletaran dan kegetaran otot (motor), serta masalah bukan pergerakan yang lain (bukan motor). Hasil beberapa kajian mencadangkan bahawa jangkitan *Helicobacter pylori* (*H. pylori*) sering berlaku di klangan pesakit PD.

***Kenapa kajian ini dibuat?*** Kami ingin mengkaji klinikal respons terhadap ubat levodopa dan gejala ‘motor’ dan bukan ‘motor’ selepas rawatan diberikan kepada pesakit Parkinson yang dijangkiti kuman *H. pylori*. Kefahaman yang lebih mendalam mengenai isu ini akan membantu meningkatkan mutu penjagaan pesakit PD.

***Apakah prosedur yang terlibat?*** Sekiranya anda bersetuju untuk menyertai kajian ini, anda dikehendaki menandatangani ‘borang keizinan penyertaan’. Semua peserta akan diberikan soalan mengenai status kesihatan, simtom abdomen dan masalah pergerakkan. Doktor terlatih akan menilai tahap keterukkan penyakit PD anda melalui penilaian klinikal. Selepas puasa semalaman, peserta akan menjalankan “urea breath test” untuk mengesan jangkitan *H. pylori*. Pesakit yang dikesan mendapat jangkitan *H. pylori* akan diberi rawatan antibiotic selama seminggu dan akan diberi dua tarikh lawat ulangan (pada 6 minngu dan 12 minggu selepas rawatan) untuk mengkaji klinikal respons terhadap ubatan levodopa dan kemajuan gejala ‘motor’ dan ‘bukan motor’ yang berkaitan dengan penyakit Parkinson menggunakan soalan kaji selidik UPDRS, PDQ 39, PD NMSQ, PD NMSS.

***Apakah risko dan keselamatan prosedur?*** Ujian “breath test” yang sering dijalankan untuk kegunaan rawatan klinikal biasa, merupakan prosedur yang mudah dan selamat.

***Apakah kebaikkan menyertai kajian ini?*** Peserta yang didapati mempunyai jangkitan *H. pylori* akan diberi rawatan antibiotik yang sesuai.

***Sulit.*** Identiti anda dan keputusan ujian anda adalah sulit.

***Anda mempunyai hak untuk menarik diri.*** Penyertaan anda adalah secara sukarela dan anda boleh menarik diri daripada kajian ini pada bila-bila masa, tanpa memberi sebarang sebab, dengan memberitahu doktor kajian yang terlibat. Sekiranya anda membuat keputusan untuk tidak menyertai kajian ini, rawatan anda akan diteruskan seperti biasa.

***Nombor untuk dihubungi.*** Sekiranya anda mempunyai sebarang persoalan, anda boleh menghubungi Dr. Hasriza Hashim, Klinik Perubatan 2, Pusat Perubatan Universiti Kebangsaan Malaysia (PPUKM), No tel: 03-91455555 extn 7300

Appendix XII(a)

CONSENT BY PATIENT FOR CLINICAL RESEARCH

I, ,…………………………………………………………………………….………………….………………….

*(Name of Patient)*

(Identity Card No: ……………………………………………………………………………………….………)

of ……………………………………………………………………………………………………………………

*(Address)*

hereby agree to take part in the clinical research specified below:

***EFFECTS OF HELICOBACTER PYLORI ERADICATION IN PATIENTS WITH PARKINSON’S DISEASE***

the nature and purpose of which has been explained to me by

Dr. ..………….………………………….…………………………………………….……………………………

*(Name & Designation of Doctor)*

and interpreted by …………………………………………..……………………..…………………………….

*(Name & Designation of Interpreter)*

to the best of his/her ability in …………………….…………… language/dialect.

I have been told about the nature of the clinical research in terms of methodology, possible adverse effects and complications (as per patient information sheet). After knowing and understanding all the possible advantages and disadvantages of this clinical research, I voluntarily consent of my own free will to participate in the clinical research specified above.

I understand that I can withdraw from this clinical research at any time without assigning any reason whatsoever and in such a situation shall not be denied the benefits of usual treatment by the attending doctors.

Date: ……………...……….. Signature or Thumbprint ……..………….………………………..………

*(Patient)*

**IN THE PRESENCE OF**

Name ………………………………………..….……..…

Identity Card No. ………………………….…… Signature…………………………....………….

(*Witness for Signature of Patient)*

Designation ……………………………….……………

I confirm that I have explained to the patient the nature and purpose of the above-mentioned clinical research.

Date ……………………………. Signature ……………………………………...…………

*(Attending Doctor)*

*DR HASRIZA HASHIM (780602-05-5026)*

| |

CONSENT BY PATIENT R.N. | |

FOR Name | |

CLINICAL RESEARCH Sex | |

Age | |

Unit | |

Appendix XII(b)

BORANG KEIZINAN OLEH PESAKIT UNTUK PENYELIDIKAN KLINIKAL

Saya…………………………………………………………………………….………………….……………...

*(Nama Pesakit)*

(No Kad Pengenalan: …...………………………………………………………………………………………)

di …………………………………………………………………………………………………………………...

*(Alamat)*

dengan ini bersetuju menyertai dalam penyelidikan klinikal disebut berikut:

***KESAN PEMBASMIAN JANGKITAN HELICOBACTER PYLORI DI KALANGAN PESAKIT PARKINSON***

yang mana sifat dan tujuannya telah diterangkan kepada saya oleh

Dr.…………………………..………………………………………………………………………………………

*(Nama & Jawatan Doktor)*

dan diterjermahkan oleh ………….…………………………………………………………………………...…

*(Nama & Jawatan Penterjemah)*

dengan sepenuh kemampuan dan kebolehannya dalam bahasa/loghat …………………………….……

Saya telah diberitahu bahawa dasar penyelidikan klinikal dalam keadaan metodologi, risiko dan komplikasi (mengikut kertas maklumat pesakit). Selepas mengetahui dan memahami semua kemungkinan kebaikan dan keburukan penyelidikan klinikal ini, saya merelakan/mengizinkan diri menyertai penyelidikan klinikal tersebut di atas.

Saya faham bahawa saya boleh menarik diri daripada penyelidikan klinikal ini pada bila-bila masa tanpa memberi sebarang alasan dalam situasi ini dan tidak akan dikecualikan daripada kemudahan rawatan oleh doktor yang merawat.

Tarikh: …………………….. Tandatangan/Cap Jari ……………………………………………….

*(Pesakit)*

DI HADAPAN

Nama …………………………………………………

No. K/P………………………………………………. Tandatangan …………*………….……………………..*

*(Saksi untuk Tandatangan Pesakit)*

Jawatan …………..………………………………….

Saya sahkan bahawa saya telah menerangkan kepada pesakit sifat dan tujuan penyelidikan klinikal tersebut di atas.

Tarikh: …….…………………..……. Tandatangan ……………………………………………

*(Doktor yang merawat)*

*DR HASRIZA HASHIM (780602-05-5026)*

| |

No. Pend. | |

KEIZINAN OLEH PESAKIT Nama | |

UNTUK Jantina | |

PENYELIDIKAN KLINIKAL Umur | |

Unit | |

**REFERENCES:**
